# Protein Arginine Methyltransferase Inhibitors Target Multiple Stages of *Plasmodium falciparum* Parasites *In Vitro*

**DOI:** 10.1021/acsinfecdis.6c00182

**Published:** 2026-03-31

**Authors:** Daniel Opperman, Tayla Rabie, Marché Maré, Mariska Naude, Mariette van der Watt, Jessica L. Thibaud, Megan Shannon, Nicole Sanders, Judith M. Bolscher, Rianne van der Laak, Rowy Willemsen, Alfred Bronkhorst, Nonlawat Boonyalai, Marcus C. S. Lee, Lyn-Marié Birkholtz

**Affiliations:** † Department of Biochemistry, Genetics and Microbiology, 56410 University of Pretoria, Private bag X20, Hatfield, Pretoria 0002, South Africa; ‡ Institute for Sustainable Malaria Control, 56410 University of Pretoria, Private bag X20, Hatfield, Pretoria 0002, South Africa; § Department of Biochemistry, 26697 Stellenbosch University, Stellenbosch 7600, South Africa; ∥ 561119 TropIQ Health Sciences, Transistorweg 5, 6534AT Nijmegen, The Netherlands; ⊥ Division of Biological Chemistry and Drug Discovery, Wellcome Centre for Anti-Infectives Research, 3042 University of Dundee, Dundee DD1 4HN, U.K.

**Keywords:** *Plasmodium falciparum*, PRMT, arginine methylation, PRMT inhibitors

## Abstract

The ongoing rise in antimalarial drug resistance underscores the urgent need for new drug candidates that specifically target novel mechanisms. Malaria parasites employ various epigenetic strategies to regulate gene expression throughout their complex life cycle, with histone lysine acetylation and methylation being well-studied and targeted by new antiplasmodials. By contrast, arginine methylation remains poorly explored. *Plasmodium falciparum* possesses three protein arginine methyltransferases (PRMTs) that maintain a unique and combinatorial histone arginine methylation landscape. Here, we present a chemical repositioning strategy to evaluate the efficacy of known PRMT inhibitors against malaria parasites. We identified a potent compound, TC-E 5003, which is active across multiple stages of parasite development. PfPRMT1 was proposed as the most likely target of TC-E 5003, with a distinct structure–activity relationship demonstrated by TC-E 5003 analogs from a hit expansion campaign. The chemotype exhibits a clear pharmacophore that elucidates the compound’s mechanism of action. Overall, these findings open a new pathway for identifying multistage active antiplasmodial candidates targeting a novel protein family in *P. falciparum*.

## Graphical abstract

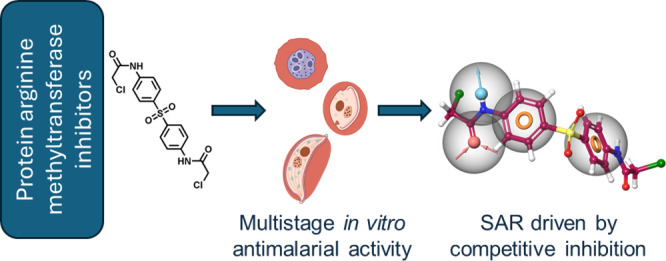

Malaria infections, which totaled 282 million malaria cases worldwide in 2024,^1^ are the result of a complex life cycle associated with the causative agent, *Plasmodium falciparum,* which survives and spreads due to adaptations across two hosts. Infection in humans begins when blood-feeding female *Anopheles* mosquitoes transmit sporozoites, which then undergo asexual exoerythrocytic development in hepatocytes. Hepatic schizonts release merozoites into the bloodstream, initiating intraerythrocytic invasion and replication during asexual schizogony, leading to massive population expansion and pathology. Parasite survival depends on continued transmission, in which <10% of asexual parasites divert to sexual development, forming transmission-ready stage V male and female gametocytes. Following transmission, male micro- and female macrogametes fuse to produce zygotes, which develop further within the mosquito, enabling the next generation of sporozoites to complete the life cycle.

The *P. falciparum* parasite exhibits highly nuanced transcriptional control that enables progression through this life cycle,^2^ achieved through unique regulatory mechanisms, including a diminished family of transcription factors compared to mammalian cells^3,4^ and a histone post-translational modification (PTM) landscape that, while sharing several conserved marks with higher eukaryotes, possesses distinct regulatory function and genomic distribution and regulates chromatin dynamics.^5–11^ The genome of asexual blood stage (ABS) *P. falciparum* parasites has a largely transcriptionally permissive euchromatic structure marked by H3K9ac and H3K4me3,^11,12^ with perinuclear heterochromatic islands marked by H3K9me3.^2,13^ Conversely, gametocytogenesis in *P. falciparum* is characterized by a more heterochromatic chromatin structure, where global gene expression reprogramming occurs through the deposition of repressive marks (for example, H3K36me2 and H3K36me3), which enacts a transcriptional shift from early gametocyte differentiation to the development of intermediate gametocyte stages.^6^

While the importance of epigenetic regulation has become increasingly evident, the role of arginine methylation in these processes remains unclear. Several arginine PTMs are present in the parasite epigenome, including H3R17me, the ubiquitously present H3R17me2 ^14,15^ and H3R8me, H3R8me2, H3R26me, H3R40me, and H3R42me.^16,17^ The occupancy of these marks is highly dynamic throughout the parasite’s life cycle, and they exhibit high connectivity, especially during gametocytogenesis.^17^ Histone and nonhistone arginine methylation PTMs are deposited by protein arginine methyltransferases (PRMTs), of which only three have been described in *P. falciparum*: *Pf*PRMT1, *Pf*CARM1/*Pf*PRMT4, and *Pf*PRMT5.^18^ This is a much-reduced protein family compared to the larger homologous families of nine PRMTs in mammals, but similar reduced family structures are also present in other protists, including *Entamoeba* and *Trypanosoma,* which each have five PRMTs.^19^ PRMTs are classified into four types according to the modifications conferred, including mono- (type III) vs. dimethylation (types I and II) and asymmetric (type I) vs. symmetric (type II) methylation, with only type I (*Pf*PRMT1 and *Pf*CARM1) and type II (*Pf*PRMT5) annotated in the parasite’s genome.

Inhibition of PRMT activity has been evaluated as an antineoplastic strategy in humans,^20–25^ with GSK3368715,^26^ Onametostat,^27^ and GSK3326595 ^28^ emerging as clinical candidates. Given the apparent importance of arginine methylation marks in several stages of *P. falciparum* development and differentiation, we evaluated whether PRMT activity can be effectively targeted in these parasites. We aimed to identify potential inhibitors of the three PRMTs described to date in *P. falciparum* parasites. Onametostat has been described to have activity against ABS parasites^29^ but was included here because its effect on other life stages of the parasite is not known. Our data identified TC-E 5003 (bis­((4-chloroacetylamino)­phenyl)­sulfone) as a potent inhibitor with activity against multiple stages of the parasite, including gametocytes, consistent with the importance of arginine methylation in both proliferation (ABS proliferation) and differentiation (gametocytogenesis). By linking the stage-spanning phenotypic effects of TC-E 5003 to the biochemical inhibition of *Pf*PRMT1, we identified TC-E 5003 as a promising chemical probe for dissecting arginine methylation biology in *P. falciparum* and strengthening the evidence for *Pf*PRMT1 as a druggable target.

## Results

### Inhibitors of Mammalian PRMTs Are Active against Multiple Stages of *P. falciparum* Parasites

A panel of known PRMT inhibitors was compiled to evaluate their ability to target multiple life stages of *P. falciparum in vitro*. Because of the considerable divergence of *P. falciparum* PRMTs compared to the human orthologs, the panel was constructed to be as diverse as possible, with inhibitors of PRMT1, PRMT3, CARM1, PRMT5, PRMT6, and PRMT7, as well as pan-PRMT inhibitors (GSK3368715, AMI-1, and MS023 targeting type I PRMTs (PRMT1/3/4/6/8), and dual-active inhibitors DS-437 (PRMT4 and PRMT7) and MS049 (PRMT4 and PRMT6) (Figure 1, Supplementary Figure S1). Of these classes, several displayed appreciable activity (>70% inhibition at 20 μM, Figure 1) against drug-sensitive *Pf*NF54 ABS parasites, including TC-E 5003 and CHEMBL2171189 (PRMT1 inhibitors), TP-064, SGC2085, and EZM 2302 (PRMT4/CARM1 inhibitors), and Onametostat, HLCL-61, and CMP5 (PRMT5 inhibitors) (Figure 1A). Interestingly, the PRMT7 inhibitor SGC3027 also showed moderate activity against ABS parasites despite the absence of an annotated *P. falciparum* ortholog. Evaluation of the activity of the PRMTi against additional life cycle stages of the parasite indicated that several of these inhibitors (TC-E 5003, MS023, TP-064, SGC2085, HLCL-61 and SGC3027) retained some activity against immature (stage II/III) gametocyte stages, but only TC-E 5003 showed appreciable activity against late (stage IV/V) gametocyte stages (Figure 1A). Overall, enrichment was observed with PRMT4/CARM1 inhibitors, with the highest levels of inhibition and appreciable activity, even at 4 μM. Among the other classes, only Onametostat (a PRMT5 inhibitor) and TC-E 5003 (a PRMT1 inhibitor) were active at this concentration. The pan-PRMT inhibitor MS023 and the PRMT4&7 dual-active inhibitor DS-437 also showed activity, whereas MS049, a dual PRMT4&6 inhibitor, did not. Orthogonal evaluation of the activities of the compounds against HepG2 cells (at 2 μM and 20 μM) indicated that only MS023 (pan-PRMT inhibitor), SGC2085 (CARM1 inhibitor), and GSK591 (PRMT5 inhibitor) showed >50% inhibition at 2 μM (Figure 1A).

**Figure 1:**
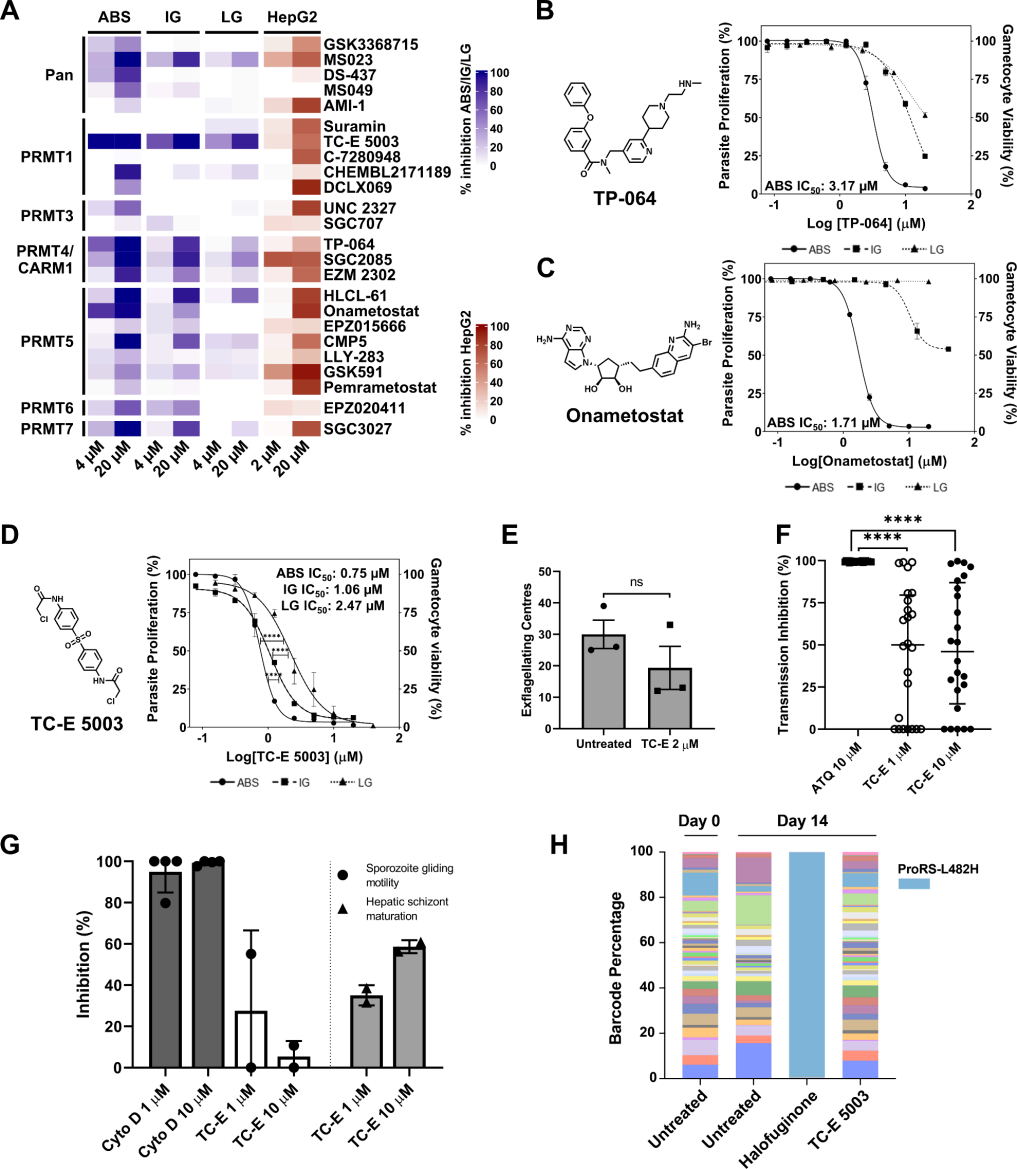
Protein arginine methyltransferase inhibitors are active against *P. falciparum* parasites. (A) Primary screen against asexual blood stage parasites (ABS, *P. falciparum* NF54 strain, 96 h of drug pressure using a SYBR Green I-based fluorescence assay) and against immature and late-stage gametocytes (IG and LG, *P. falciparum* NF54-*pfs*16-GFP-Luc strain, 48 h of drug pressure using a luciferase reporter assay) and against HepG2-A16-CD81 hepatocellular carcinoma (48 h of drug pressure, LDH cytotoxicity assay). (B–D) Dose–response against asexual (solid line, closed circles, ABS, 96 h of drug pressure), immature gametocyte (coarse dashed line, closed squares, IG, 48 h of drug pressure) and late-stage gametocytes (fine dashed line, closed triangles, LG, 48 h of drug pressure) treated with (B) TP-064, (C) Onametostat or (D) TC-E 5003. Extra sum-of-squares F test, *P* < 0.0001. Data are from 3 biological repeats (*n* = 3), ± SEM (E) Inhibition of male gamete exflagellation from three biological repeats (*n* = 3), mean ± SEM. Mann–Whitney test, *P* = 0.4 (F) Decrease in oocyst intensity by TC-E 5003 compared to an atovaquone (ATQ) control. Data indicate the interquartile range. Kruskal–Wallis statistical test, *P* < 0.0001. (G) Inhibition of sporozoite gliding motility and hepatic schizont maturation (H) Average barcode population counts from the AReBar assay, comparing day 0 population diversity (by colors) to day 14. Expansion of ProRS-L482H resistant line for the positive control treatment (halofuginone). ProRS, proline tRNA synthetase.

Subsequent full dose–response evaluation indicated that both TP-064 (a PRMT4/CARM1i) and Onametostat (a PRMT5i) inhibited asexual stages of both drug-sensitive and -resistant *P. falciparum* lines (*Pf*Nf54 vs. *Pf*Dd2) with IC_50_s of 3.17 ± 0.21 μM against *Pf*NF54 (1.53 ± 0.56 μM against *Pf*Dd2) and 1.71 ± 0.062 μM (1.15 ± 0.20 μM against *Pf*Dd2), respectively (Figure 1B,C, Supplementary Figure S2A,B). However, these compounds were poorly active against IG and LG at concentrations of 20 μM (Figure 1B,C). Both compounds were more active against the parasites than against mammalian cells, with >80-fold selectivity with CC_50_s of >20 μM for TP-064 against both HepG2 and Chinese hamster ovary (CHO) cells (Supplementary Figure S2D) and of 143.81 ± 110.02 μM and 135.09 ± 79.71 μM against HepG2 cells and CHO cells, respectively, for Onametostat, reflecting selectivity values of 85- and 79-fold (Supplementary Figure S2E).

By contrast, TC-E 5003, as a PRMT1 inhibitor, exhibited appreciable activity against the asexual stages of both *Pf*NF54 and *Pf*Dd2, with IC_50_ = 0.75 ± 0.055 and 0.37 ± 0.111 μM, respectively (Figure 1D, Supplementary Figure S2A). Importantly, the dose–response evaluation showed that TC-E 5003 also showed comparable activity (<2-fold loss) against immature gametocytes (IC_50_ = 1.06 ± 0.001 μM) and appreciable activity against late-stage gametocytes (IC_50_ = 2.47 ± 0.354 μM) (Figure 1D). This 3-fold change in activity between ABS and late-stage gametocytes (*P* < 0.0001, extra sum-of-squares F test, n = 3 ± S.E.M.) is similar to that seen for clinical candidates such as M5717 and could equate to gametocytocidal efficacy given reduced gametocyte population densities.^30^ TC-E 5003 treatment reduced male gamete exflagellation by 28% compared to the untreated control (Figure 1E, *P* = 0.4) and further resulted in a 50% decrease in oocyst intensity at 1 μM (Figure 1F), indicating some transmission-blocking potential. However, this compound has little to no effect on sporozoites or hepatic schizonts with only 35% inhibition of the latter (Figure 1G), indicating that it would not have prophylactic or preventive efficacy. The multistage activity was associated with parasite selectivity, with TC-E 5003 17× and 24× more active against *P. falciparum* parasites than mammalian HepG2 and CHO cells, respectively (CC_50_s of 7.05 ± 1.2 and 17.94 ± 3.8 μM; Supplementary Figure S2F).

Evaluation of TC-E 5003 in the antimalarial resistance barcoding (AReBar) assay^31^ further revealed no recrudescence of parasites containing 50 different resistance mutations (Figure 1H, Supplementary Figure S3, Supplementary Table S1), indicating that single nucleotide polymorphisms associated with drug resistance do not confer resistance to TC-E 5003, and potentially supporting a novel mode-of-action not associated with currently characterized antimalarial drug targets and their resistance profiles. Taken together, these data identify inhibitors of mammalian PRMTs as having activity against ABS stages of *P. falciparum*, with TC-E 5003 as the most active and also displaying multistage activity, with a potentially novel mode-of-action.

### Treatment with TC-E 5003 and TP-064 Results in Aberrant Schizogony

We subsequently evaluated the effect of inhibition of *Pf*NF54 ABS proliferation for the three most active compounds TC-E 5003, TP-064 and Onametostat (Figure 2, Supplementary Figure S4). Treatment of ABS parasites with these compounds revealed that the parasites can progress from rings to trophozoites without any observable morphological aberrations by 24 h post-treatment (hpt). However, by 36 hpt, the trophozoites show delayed development with reduced cell growth, including punctate hemozoin crystals, which could be a result of reduced metabolic activity and hemoglobin degradation. However, TC-E 5003 was a poor inhibitor of β-hematin formation with an IC_50_ of 173.9 ± 1.044 μM compared to chloroquine at 22.4 ± 0.30 μM (Supplementary Figure S4, *P* < 0.0001, extra sum-of-squares F test), which indicates that inhibition of hemozoin formation^32^ is likely not a main contributing factor to the mode of action.

**Figure 2:**
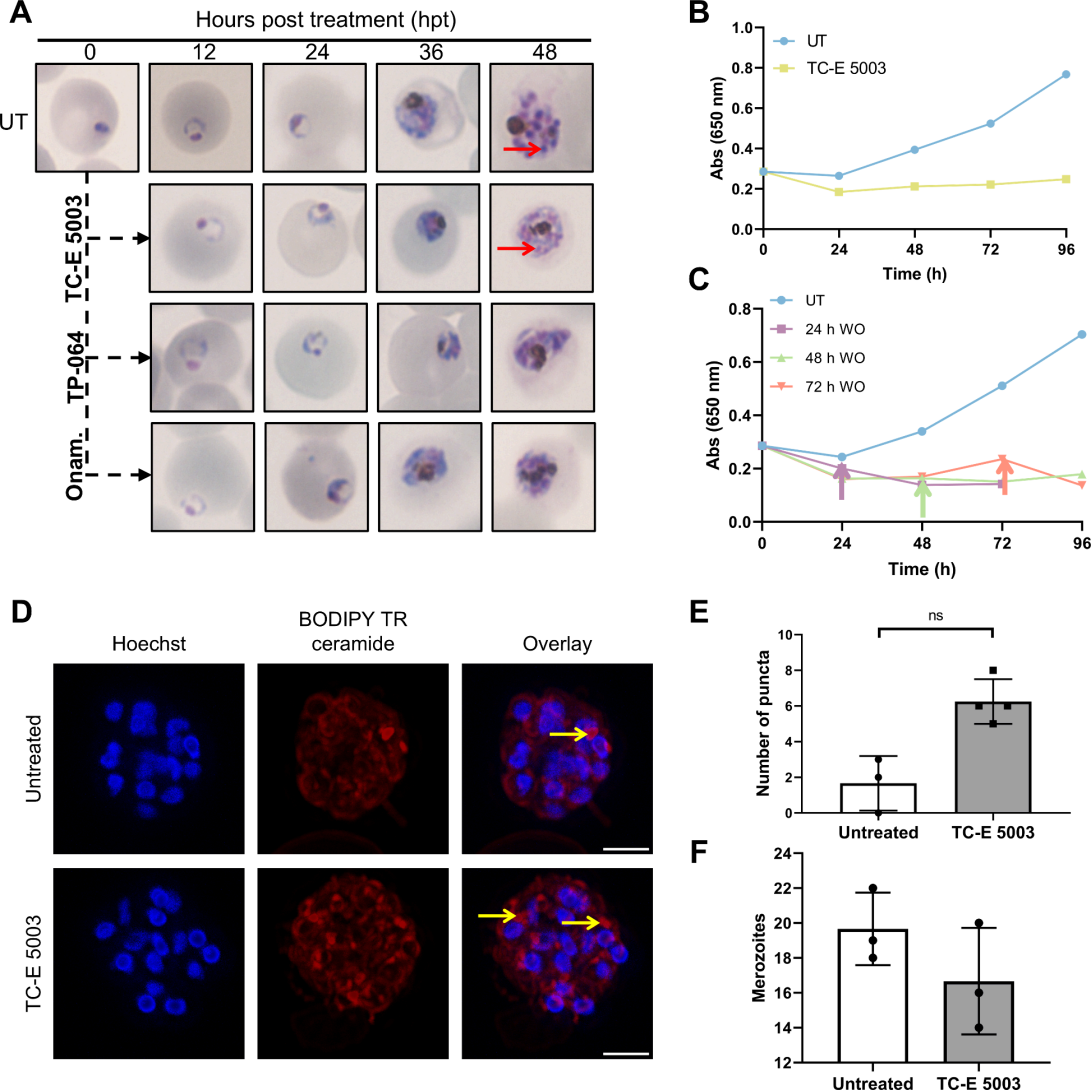
PRMT inhibitors adversely affect schizogony in malaria parasites. (A) Morphologies observed in asexual NF54 strain malaria parasites at approximately 2 hpi treated with TC-E 5003, TP-064 or Onametostat for 48 h. (B) ABS parasite viability over a 96 h treatment at 3× IC_50_. Data are from three biological repeats (*n* = 3), ± SEM (C) ABS parasite viability over 96 h with drug washout (WO) at 24, 48, and 72 h post treatment. Arrows indicate the time point of washout. Parasites treated at 3× IC_50_. Data are from three biological repeats (*n* = 3), ± SEM (D) Confocal microscopy of NF54 *P. falciparum* schizonts treated for 24 h with TC-E 5003 at 3× IC_50_. Nuclei are stained with Hoechst, and membranes are stained with BODIPY TR Ceramide. Membranous puncta are indicated with yellow arrows. Scale bars are 2 μm. (E) Number of membranous puncta in TC-E 5003-treated vs untreated. Mann–Whitney test, *P* = 0.0571. (F) Merozoite counts of 24 h TC-E 5003-treated vs untreated schizonts. Data are from *n* = 3, ± SEM.

However, after 48 h of treatment, each compound led to aberrant schizogony in the parasites. Some nuclear replication seemed present, as evidenced by nuclear staining in the parasites (Figure 2A), however, these parasites were seemingly unable to complete cellular division to form daughter merozoites, with no evidence of packaged daughter cells present. Treatment periods longer than one life cycle (up to 96 h) indicated the presence of pyknotic, compromised parasites (Supplementary Figure S5B), with decreased parasitaemia (Figure 2B). This phenotype was parasitocidal and not parasitostatic, with parasites unable to recover even after only 24 h of exposure, as removal of TC-E 5003 in a washout experiment did not result in any recrudescence of parasites (Figure 2C). The observed phenotype was evaluated in more detail with fluorescence microscopy, which indicated that TC-E 5003 treatment of 24 h postinvasion (hpi) trophozoites for 24 h indeed affected cytokinesis and daughter cell packaging (Figure 2D), with anomalous membranous puncta present, 3-fold higher that in untreated cells (Figure 2E, Mann–Whitney test, *P* = 0.0571). Nuclear replication and division were completed efficiently, as indicated by the nuclear staining pattern (Figure 2C), and the total number of nuclei did not differ appreciably from that of the untreated parasites (Figure 2F).

The time frame of the observed effect was associated with parasites at the trophozoite stage of development and only apparent upon treatment of newly infected rings through to early trophozoite stages within 24 hpi (Supplementary Figure S5). Treatment past this window on mature trophozoites (36 hpi) did not affect the parasites and resulted in normal schizogony. These data correlate with the expected availability particularly of *Pf*PRMT1, which shows a peak in transcript production in parasites at 20–26 hpi, correlating to protein availability^33^ (Supplementary Figure S6). This protein is also expressed in gametocyte stages. Comparatively, neither PfPRMT4 nor PfPRMT5 has a similarly distinct expression profile with a clear peak in ABS parasites, and may therefore be required by the parasite in lower quantities, with *Pf*PRMT5 particularly implicated in merozoite invasion and RNA splicing.^34^ Additionally, *Pf*PRMT1 expression in gametocytes correlates with the phenotypic activity profile, with both transcript and protein present in both immature and mature gametocytes.^35^ These data point to *Pf*PRMT1 as the most likely PRMT family member to be a candidate for inhibition by TC-E 5003.

### TC-E 5003 Inhibits Recombinant *Pf*PRMT1

To understand how TC-E 5003 exerts its activity, molecular modeling and docking were used to assess its potential binding to *P. falciparum* PRMT1. A homology model of the *Pf*PRMT1 functional dimer was generated from the human orthologue *Hs*PRMT1 (PDB: 8Z2Z), which shared 46% sequence identity with a backbone RMSD of 0.151 Å and with complete coverage of the catalytic fold (Figure 3). *Pf*PRMT1 contained all the characteristic PRMT1 domains (Supplementary Figure S7), including a conserved dimerization domain and core catalytic domain with a YFxxY motif, double-E loop, I, pI, II, III, and a THWxQ motif.

**Figure 3:**
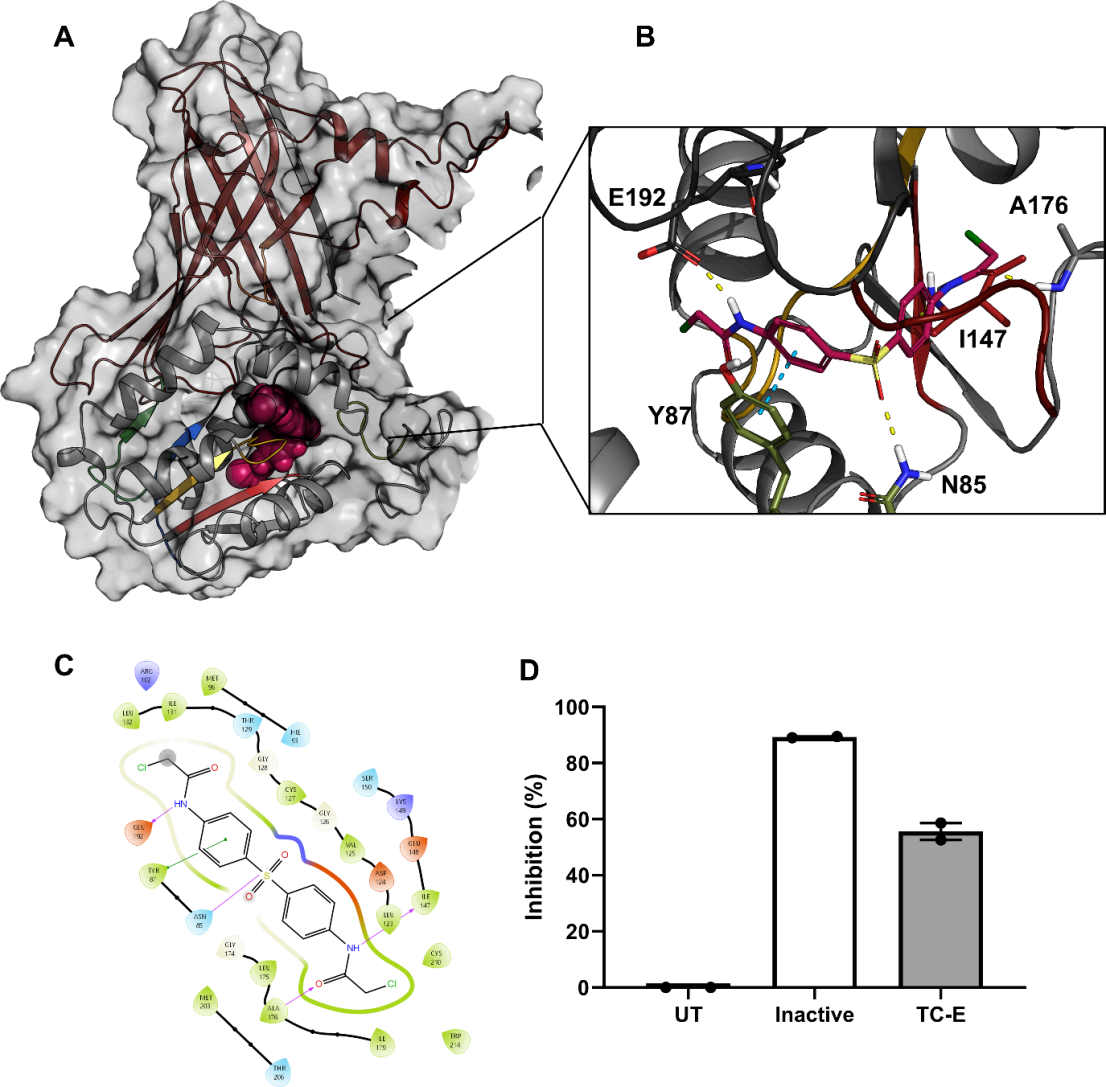
TC-E 5003 inhibits the *Pf*PRRMT1. (A,B) TC-E 5003 docked into the homology model of *Pf*PRMT1. TC-E 5003 is indicated in magenta. Dimerization domain is shown in deep burgundy with characteristic motifs YFxxY in olive green, I in gold, pI in red, II in blue, double E loop in black, III in green, and THW in burnt orange. (A) full view, (B) zoomed view. (C) Key interactions between TC-E 5003 and PfPRMT1. Hydrophobic residues are shown in green, glycine in yellow, negative in red, and positive in blue. Hydrogen bonds are indicated in purple, and π–π stacking interactions are indicated in green. Gray clouds around ligand atoms indicate solvent-exposed regions. (D) Inhibition of recombinant *Pf*PRMT1 by TC-E 5003. Data are from two biological repeats (*n* = 2) performed in technical duplicate, ±SEM. UT: untreated; inactive: protein denatured by boiling; TC-E: TC-E 5003.

Induced-fit docking of TC-E 5003 (Figure 3A) revealed the occupation of the *S*-adenosyl-l-methionine (SAM) binding pocket. The compound forms hydrogen bonds with the polar hydrogens of N85 and A176 (Figure 3B). A π–π stacking interaction also forms between a phenyl group and Y87 of the conserved YFxxY motif, which stabilizes the *S*-adenosyl-l-homocysteine (SAH) cosubstrate adenine ring.^36^ Furthermore, the compound interacts with E192 via a hydrogen bond within the double E loop, which positions the substrate arginine (Figure 3C). A subsequent 20 ns molecular dynamics simulation performed using Desmond also revealed a hydrogen bond between H93 and a chloroacetamido nitrogen, a π-cation interaction between a phenyl group and K149, and a water-mediated hydrogen bond between E201, which is part of the double E loop and coordinates the substrate arginine. This binding mode is largely recapitulated by TC-E 5003 when docked to human PRMT1 (Supplementary Figure S8), with the hydrogen bond between TC-E 5003 and the double E loop glutamic acid E162 retained, supporting a potentially similar mechanism of inhibition between the two proteins. While chloroacetamide moieties are well-characterized covalent bonding warheads and have been suggested to covalently bind *Hs*PRMT1 C119 through alkylation,^37^ empirical data that support this mechanism of action are still needed.

To confirm that TC-E 5003 inhibits *Pf*PRMT1, the protein was recombinantly expressed, and its inhibition by TC-E 5003 was evaluated *in vitro* (Supplementary Figure S9). The identity of the protein was confirmed by using TIMs-TOF mass spectrometry. Consistent with predicted binding, TC-E 5003 inhibited recombinant *Pf*PRMT1 by 56 ± 3% at 10 μM (Figure 3D). These data indicate a mechanistic and functional similarity between the inhibition of *Pf*PRMT1 and its human homologue, *Hs*PRMT1 by TC-E 5003.

### Hit Expansion and Structure–Activity Relationship of TC-E 5003 Analogs

Analogs of TC-E 5003 were subsequently evaluated to clarify the binding mode, identify the minimal pharmacophore required for *Pf*PRMT1 inhibition and to assess tolerance to structural changes (Table 1; Supplementary Figure S10). Matched-pair analysis revealed similar (∼2-fold change) *in vitro* whole-cell activities against ABS *Pf*NF54 parasites for the sulfonyl (TC-E 5003, IC_50_ = 0.75 μM) and sulfide linkers (An16, IC_50_ = 1.4 μM). The bis­(phenyl) framework of the compound drives activity since a 10-fold loss in activity is present when the symmetry is lost in truncated versions, as in An22 (IC_50_ = 7 μM). This clearly supports the binding mode of TC-E 5003 within the SAM pocket, requiring the spatial orientation of the phenyl moieties to span the channel, allowing interaction and stabilization with Y87 on one end and K149 on the other. Indeed, the 2-chloro moieties are necessary for activity in both the sulfones and sulfides, with removal on both ends (as in, e.g., An11 and An17 for the sulfones/sulfides, respectively), rendering them inactive, but singular removal (e.g., An14 and An15) retains some activity.

**Table 1:**
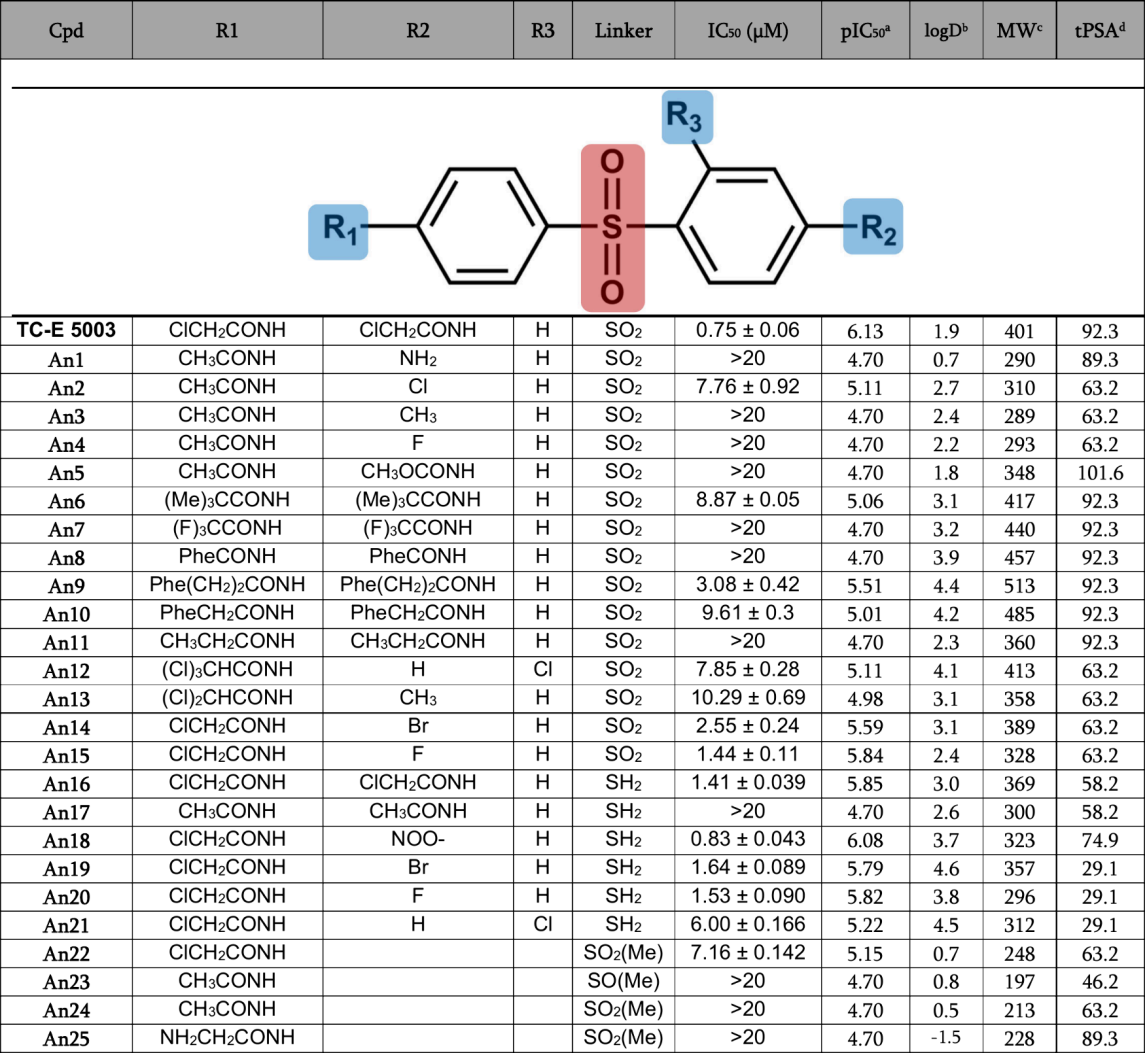
Properties and Antiplasmodial Activities of TC-E 5003 and Analogs^a^

* Data include activity (pIC_50_).

a pIC_50_, −log_10_(IC_50_[M]).

b logD, distribution coefficient.

c MW, molecular weight.

d tPSA, topological polar surface area.

Subsequent SAR expansion for the series to understand the contributors of TC-E 5003 to activity against *P. falciparum* parasites indicated a dependence on substitution at the western end of the compound (Table 1). Analogs that retained the chloroacetamido moiety on the western side, but where the eastern side moiety was reduced to either F or Br (e.g., An14 and An15), only displayed a 2–3-fold loss in activity. Reducing the chloroacetamido group to an acetamido group at the western end resulted in a complete loss of activity, irrespective of the change at the eastern end (as in An3, An4). This indicates that a strong electron-withdrawing group is required at the terminus of the western end, with An11 showing a > 10-fold loss compared to TC-E 5003. The SAR trends remain true for the sulfide-linked derivatives, with activity driven by the presence of the chloroacetamido group at the western end of the molecule, irrespective of changes on the eastern side (An18, An19, An20). More bulky substitutions at both ends are only somewhat tolerated for a phenethyl (An9, 4-fold loss in activity), with a more dramatic loss in activity for benzyl or phenyl substitution (An10, An8), as is true for a *tert*-butyl bisubstitution (An6). All other examples of compounds where the symmetry was lost were completely inactive (An23, An24, and An25).

Based on this information, a pharmacophore for TC-E 5003 was developed using PHASE,^38^ defining the minimum set of features required for activity within the congeneric TC-E 5003 analogue panel (Figure 4). This confirmed that the minimum features required for potent activity (IC_50_ ≤ 2 μM) include both aromatic rings, and on the western side of the molecule, a hydrogen bond donor and acceptor and hydrophobic moiety (Figure 4A). The electron-withdrawing presence of a terminal Cl strongly influences the hydrogen bond donor capacity of the amide nitrogen, which is particularly important for its hydrogen bonding on the western end to Glu192 (Figure 4B). Validation of the model revealed a complete match of the pharmacophore to each of the six active compounds, with partial matches for all but two of the inactive compounds, which matched fully. Enrichment analysis using 800 decoys from DUD-E, with physicochemical properties similar to those of the actives, confirmed the model’s specificity, with all six active compounds ranked above the decoys (BEDROC160.9 = 1.00, ROC = 1.00). The minimum pharmacophore reveals that, aside from the required two sequential aromatic rings, a hydrogen bond between E192 and the hydrogen bond donor as well as between H93 and the hydrogen bond acceptor is essential for activity, with the requirement of a hydrophobic moiety, such as chlorine, in close proximity.

**Figure 4:**
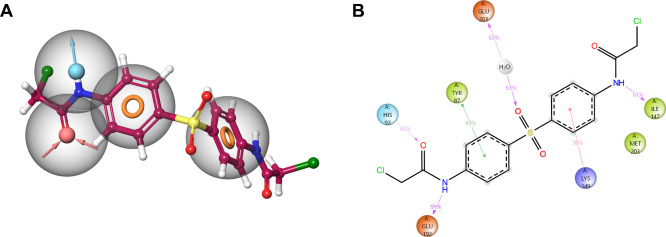
SAR and pharmacophore of TC-E 5003. (A) Pharmacophore of TC-E 5003 and its analogs with TC-E 5003 overlaid. Aromatic ring features are shown in orange, and the hydrogen bond donor is shown in blue and the hydrogen bond acceptor in red, with possible hydrogen bond vectors shown as arrows. (B) Ligand protein contacts over 20 ns molecular dynamics simulation with contact strength indicated. Positive residues shown in blue, negative in red, hydrophobic in green, histidine in light blue, and water in gray. Hydrogen bonds are shown in purple, π–π stacking interactions in green, and π-cation interaction in red.

## Discussion

Here, we demonstrate that several PRMT inhibitors previously investigated as antineoplastics exhibit activity against *P. falciparum* parasites, implicating PRMTs as a potentially targetable family within these parasites. Other organisms have proven vulnerable to PRMT inhibitors; treatment of *Toxoplasma gondii* with AMI-1, a pan-PRMT inhibitor that does not compete with SAM,^39^ inhibited *Tg*CARM1 but not *Tg*PRMT1 and resulted in decreased H3R17me2 occupancy and induction of cyst formation in tachyzoite stages.^40^ AMI-5, a general arginine and lysine methyltransferase inhibitor, has demonstrated activity against recombinantly expressed *Pf*PRMT1, as has the SAH analogue Sinefungin.^18^ These findings underscore the amenability of *P. falciparum* arginine methylation processes to drug targeting. With PRMTs involved in histone PTMs and the parasite’s known sensitivity to epigenetic modulators,^41^ histone arginine methylation appears particularly important across multiple stages of the parasite’s development, including gametocytes.^17^ However, targeting PRMTs in *P. falciparum* may provide advantages associated with pleiotropic modes-of-action, since PRMTs are involved in methylating arginine on substrate proteins involved in multiple complex processes (including DNA damage responses and RNA metabolism) and not simply histone arginine methylation.^41–48^

Our chemical interrogation strategy sheds light on which of the PRMT family members show the most druggable promise. Onametostat targets *Pf*PRMT5 and decreases histone H3R2me2s,^29^ and our data show that this compound is the only example of a PRMT5 inhibitor with modest but selective activity toward ABS parasites. This correlates with *Pf*PRMT5 knockout data, which show decreased merozoite invasion of erythrocytes, consequent to decreased deposition of the activating mark H3R2me2s, particularly on invasion-related genes in ABS parasites.^34^ *Pf*PRMT5 may therefore only play a role in controlling histone arginine methylation in ABS parasites. This may also be true for *Pf*PRMT4/CARM1, since its potential inhibition by TP-046 could alter the levels of H3R17me2, an activating mark. This histone PTM is depleted from transcriptionally silenced genes in ring stages but is enriched in gene bodies of female gametocyte genes,^49^ and coordinates with H3R8me2s in both immature and mature gametocytes.^17^ Given TP-064’s activity profile, targeting both ABS and immature gametocytes, the involvement for *Pf*PRMT4 in epigenetic modulation at these stages is plausible.

However, the only family member that shows potential as a target across multiple stages of malaria parasite development is *Pf*PRMT1. TC-E 5003, as a proposed *Pf*PRMT1 inhibitor, elicited potent submicromolar multistage activity against both ABS parasites and gametocytes. TC-E 5003 shows particular selectivity for *Hs*PRMT1, as it inhibits recombinant *Hs*PRMT1 with an IC_50_ of 1.5 μM without affecting *Hs*PRMT4, with only ∼15% inhibition of this protein at 500 μM of TC-E 5003.^50^ This preference toward *Hs*PRMT1 translates to cellular activity, and TC-E 5003 shows activity against MCF7a breast cancer lines, LNCaP prostate cancer cell lines and also blocks androgen-dependent transcription in LNCaP cells, consistent with PRMT1’s role in activating androgen receptors in mammalian cells.^50^

*Pf*PRMT1 has previously been demonstrated to possess canonical PRMT1 activity, methylating H4R3, a PTM involved in transcriptional activation, *in vitro.* ^18^ PRMT1 in the related apicomplexan parasite *T. gondii* has been thoroughly linked to cell cycle control, with loss of *Tg*PRMT1 activity disrupting centrosome dynamics and microtubule organizing center (MTOC) function, resulting in failure to segregate progeny, aberrant daughter buds, and loss of replication synchronicity.^51^ This is consistent with the failure to complete schizogony in *P. falciparum* observed upon TC-E 5003 treatment. *P. falciparum* parasites, like *T. gondii*, critically rely on centrosome and MTOC function,^52,53^ with the mitotic machinery also implicated in gametocyte development through involvement in chromatin reorganization and characteristic cellular elongation.^54^ However, RNA metabolism could also be affected as a nonhistone mediated dysregulation based on TC-E 5003 treatment, since transcriptional processes are active in mature gametocytes to enable a poised environment for onward transmission.^35^ *Pf*PRMT1 localizes to the nucleus and cytosol, supporting its role in methylation of nonhistone proteins involved in RNA metabolism, including fibrillarin as an RNA-binding protein, the polyadenylation protein PABPII, rpS2 as a component of the 40S ribosomal subunit, and the splicing factor SR1.^18^

Our data show that TC-E 5003 can inhibit *Pf*PRMT1 by binding to the SAM binding site. *Hs*PRMT1 has been suggested to be alkylated at C119 at the pI domain by TC-E 5003,^37^ although not empirically validated. This residue is replaced by a lysine (K149) in *P. falciparum*, and no other cysteine is conserved between *Pf*PRMT1 and *Hs*PRMT1 within the binding pocket, which would be consistent with covalent bonding by alkylation in the Plasmodium protein. Rather, the SAR that is evidently driving TC-E 5003’s activity and the resultant pharmacophore support that the activity of TC-E 5003 could be associated with coordinating into and occluding the SAM site of *Pf*PRMT1. However, biochemical confirmation of these important changes in head-to-head comparisons of the inhibition of *Hs*PRMT1 and *Pf*PRMT1 (and indeed other PRMT family members) is needed to identify improved small-molecule inhibitors with selectivity toward the *P. falciparum* protein.

Although these data indicate that PRMT inhibitors clearly affect multiple stages of *P. falciparum* development, direct *in situ* target engagement and specificity toward *Pf*PRMT1 with, e.g., quantitative chemical proteomics is needed to confirm the causal link between the phenotypic effects of TC-E 5003 against *P. falciparum* parasites *in vitro* and the targeting of *Pf*PRMT1 *in situ*, as demonstrated with recombinantly expressed *Pf*PRMT1. Additional target validation (with, e.g., conditional knockdown) is further needed to confirm *Pf*PRMT1 as the sole target, since the current data cannot exclude the involvement of any additional “off-target” effects. Moreover, the druggability of *Pf*PRMT1 needs thorough validation,^55^ as initial PiggyBac transposon mutagenesis for *P. falciparum* indicated that this protein is dispensable,^56^ whereas high-coverage transposon mutagenesis in *P. knowlesi* shows it is essential,^57^ and direct gene disruption in *P. berghei* resulted in a clear growth defect.^58^

The characterization of several PRMT inhibitors in this body of work, therefore, showed the potential of *P. falciparum* protein arginine methylation and PRMTs as a targetable biology in *P. falciparum*.

## Conclusion

Our data demonstrate the potential of histone arginine methylation as a targetable process in *P. falciparum* parasites. It has become increasingly evident that histone arginine methylation is an important regulatory mechanism throughout the parasite lifecycle, with recent publications demonstrating the coordinated deposition of these PTMs and their role in regulating important cellular processes. It is therefore apparent that further characterization of histone arginine methylation is a priority and that these processes stand to be exploited as targets for chemical inhibition. Parasite protein arginine methylation is a burgeoning field, and the identification of *P. falciparum* protein arginine methyltransferase inhibitors will enable the exploration of these fundamentally important processes and facilitate the development of new chemical matter to exploit these processes in *P. falciparum* parasites.

## Methods

### *In Vitro* Cultivation of *P. falciparum* Parasites

Asexual stage *P. falciparum* parasites of the drug sensitive NF54 strain, the chloroquine, pyrimethamine and mefloquine resistant Dd2 strain, as well as NF54-*pfs16*-GFP-Luc and 3D7elo1*-pfs16*-CBG99 luciferase reporter lines^59^ were cultivated *in vitro* and maintained in human erythrocytes suspended in complete RPMI 1640 medium, synchronized to the ring stage. Blood was obtained from human donors (after obtaining informed consent) or from the South African National Blood Service and was covered under ethical approval from the Health Sciences Ethics Committee (506/2018) and the Natural and Agricultural Sciences Ethics Committee (180000094) of the University of Pretoria.

### Activity Assay for Asexual Blood Stages Proliferation

A protein arginine methyltransferase inhibitor panel was screened against *P. falciparum* asexual blood stage parasites using a SYBR Green I-based proliferative assay at 1% hematocrit and 1% parasitemia for 96 h, under appropriate atmospheric conditions (5% CO_2_, 5% O_2_, 90% N_2_) and performed in technical triplicate as before.^30^ Chloroquine disulfate was used as a drug-treated control. Primary screens were conducted at 4 and 20 μM. Fluorescence was ascertained using a Fluoroskan Ascent FL microplate fluorometer (excitation at 485 nm, emission at 538 nm). Selected compounds were progressed for full dose–response determination to ascertain the half-maximal inhibitory concentrations (IC_50_s). These were performed in a technical triplicate for three independent biological replicates.

### Viability Assay of Gametocyte Stages

Gametocytes were induced from asexual NF54-*pfs16*-GFP-Luc^60^ or 3D7elo1-*pfs*16-CBG99 lines.^61^ The PRMTi panel was screened against immature (>90% stage II/III) and late-stage (>90% stage IV/V) gametocytes with a luciferase reporter assay at a 2% hematocrit and 2% gametocytemia using methylene blue and the clinical candidate MMV390048 as drug-treated controls, as before,^62^ with 48 h of drug pressure. Luminescence was determined in white plates by addition of 30 μL of Luciferase Assay System substrate (Promega) to 30 μL of gametocyte culture (NF54-pfs16-GFP-Luc) or 100 μL of a nonlysing substrate d-luciferin substrate (1 mM in 0.1 M citrate buffer, pH 5.5) to 100 μL of gametocyte culture (CBG99) with an integration constant of 2 s on a GloMax Multi detection system. Inhibition was normalized to minimal inhibition of untreated controls and the IC_50_ was obtained by nonlinear curve fitting (GraphPad Prism 8).

### Evaluation of Male Gamete Exflagellation Inhibition by TC-E 5003

Inhibition of male gamete exflagellation was measured^30^ by treating mature, stage V gametocytes with TC-E 5003 at 2 μM, with an untreated control and a drug-treated control with 10 μM methylene blue. Cultures were incubated for 48 h under hypoxic conditions, 1 mL of each culture was harvested by centrifugation, and the pelleted parasites were resuspended in 50 μL of ookinete medium (glucose-enriched medium supplemented with 100 μM xanthurenic acid and 50% (v/v) human serum). Suspensions were incubated for 8 min, and then 10 μL was loaded onto a hemocytometer and incubated for a further 8 min at room temperature. Exflagellation centers were recorded by videography every 30 s for 8 min with a 10× objective and enumerated semiautomatically using Icy Bioimage Analysis Software.

### Counter-Screening for Cytotoxicity

HepG2-A16-CD81 hepatocellular carcinoma or Chinese Hamster Ovary (CHO) cells were seeded into a 96-well plate at a density of 1 × 10^5^ or 1 × 10^4^ cells per well, respectively, in 100 μL of Dulbecco’s modified Eagle’s medium and allowed to adhere and proliferate for 24 h. The medium was then aspirated and replaced with fresh medium containing a 2-fold serial dilution of compounds to be tested, with emetine as a drug-treated control. Plates were incubated at 37 °C for a further 48 h. Inhibition was then colorimetrically determined by either LDH or MTT activity. For LDN, 90 μL of supernatant from each well was added to 10 μL of CytoSelect LDH Cytotoxicity Assay Reagent (Cell Biolabs Inc.), followed by 1 h of incubation under hypoxic conditions at 37 °C and measurement of absorbance at 450 nm. For MTT, 25 μL of 3-(4,5-dimethyl-2-thiazolyl)-2,5-diphenyl-2H-tetrazolium bromide (MTT) was then added to each well and incubated for a further 4 h and centrifuged to collect formazan crystals, which were dissolved in 100 μL of DMSO per well with agitation. Absorbance was determined at 540 nm.

### Antimalarial Resistome Barcode Sequencing (AReBar) Cross-Resistance Assay

Cross-resistance of the compounds was assessed against a pool of 52 barcoded drug-resistant parasites^31^ (Supplementary Table S1) in both the Dd2 and 3D7 backgrounds. These lines were barcoded at the *pfpare* safekeeping locus (PF3D7_0709700)^63^ with unique 11-bp barcode sequences. Triplicate cultures (1 mL each in the pool) were exposed to 3× IC_50_ of each compound, including the positive control halofuginone, which targets prolyl tRNA synthetase. Parasitemia was monitored every 2–3 days by flow cytometry (Beckman CytoFlex 5), with 1x SYBR Green I and 200 nM MitoTracker Deep Red staining, maintaining parasitemia below 5%. On day 14, parasites were isolated with 0.05% (w/v) saponin, and the barcodes were amplified with PCR for quantification with Oxford Nanopore minION sequencing. Barcode proportional changes were indicated as log_2_ fold change relative to the no-drug control, with values >2.5 indicating cross-resistance.

### Morphological Evaluation of Parasites to Determine the Effect of Inhibition on Parasite Development

Asexual early ring-stage NF54 strain parasites were synchronized to the ring stage using 5% sorbitol and treated at 3× IC_50_ with TC-E 5003, TP-064 or Onametostat and monitored for 48 at 12 h intervals using Giemsa smears. Parasite morphology was evaluated under a light microscope at 100× magnification with oil immersion.

### Liver Stage Activity

Cryopreserved human primary hepatocytes (PHHs) were thawed and seeded at 18,000 cells per well in collagen-coated 384-well plates and incubated at 37 °C with 5% CO_2_, medium refreshed 24 h later and 48 h after plating, freshly dissected *P. falciparum* NF54 sporozoites (25,000 per well) were transferred onto the cells. Plates were centrifuged for 10 min at 1900 × g and incubated at 37 °C with 5% CO_2_ for 3 h after which sporozoites were aspirated. Compounds (diluted in culture medium) were added to the hepatocytes, and the medium containing the compounds was refreshed daily for 4 days. After this, cells were fixed with ice-cold methanol, washed three times with phosphate-buffered saline (PBS)-Tween20 (PBS-T), and stained with anti-*Pf*HSP70 antibody for 1 h at RT. After three washes with PBS-T, cells were costained with DAPI and antirabbit secondary antibody for 1 h at RT, washed 3× and imaged on an ImageXpress PICO automated imaging system. The total number of cells and the number of invaded cells were quantified using CellReporterXpress software. Inhibition of infection was calculated relative to wells where sporozoites were incubated in medium alone.

### Screening of Sporozoite Stages

Optical 96-well plates were coated with anti-CSP mAb 3SP2,^64^ washed and blocked with assay buffer (containing 10% heat-inactivated FBS) as described previously.^65^ Salivary gland sporozoites (spz) were isolated from *Anopheles stephensi* mosquitoes infected with *P. falciparum* in L15 Leibovitz medium and supplemented with 10% heat-inactivated FBS. Sporozoites were preincubated for 30 min with compounds at RT. Following incubation, sporozoites were transferred to the assay plate, centrifuged at 1900 × g for 10 min and allowed to glide for 90 min at 37 °C, 3% O_2_, and 4% CO_2_. Next, sporozoites were removed, plates washed, fixed using 4% (v/v) paraformaldehyde, and blocked with an assay buffer. Gliding trails were stained using a biotinylated mAb 3SP2 antibody and a streptavidin AF555 conjugate and captured by automated high content imaging using ImageXpress PICO (Molecular Devices). Total gliding trail length was analyzed using MetaXpress software with outputs as the amount of positively stained surface normalized against medium (negative) and 10 μM CytoD (positive) controls.

### Standard Membrane-Feeding Assays

Stage V gametocytes from *P. falciparum* strain NF54-hsp70-GFP-luc were fed to *An. stephensi* mosquitoes during a bloodmeal.^66^ For the direct mode of the SMFA, 200 μL of parasite culture was added to 200 μL of packed red blood cells, centrifuged for 20 s at 10,000 × g, and the supernatant aspirated. Compound (5 μL) diluted in incomplete medium was added to 195 μL of human serum type A (0.1% DMSO final) and added to the gametocyte culture pellet. The final 400 μL mixture was immediately injected into an individual membrane-covered minifeeder, where 40 female *An. stephensi* mosquitoes were allowed to feed for 10 min. Controls included vehicle (0.1% DMSO) and 10 μM atovaquone. Unfed and partially fed mosquitoes were removed from the cage after feeding, and fed mosquitoes were maintained at 26 °C and 80% humidity. Eight days postfeeding, the infection intensity (number of oocysts) and infection prevalence (percentage of mosquitoes carrying at least one oocyst) were determined by luminescence analysis of individual mosquitoes.

### Confocal Microscopy

Asexual parasites (NF54 strain) at approximately 20 hpi were treated for 24 h at 3× IC_50_ and seeded onto coverslips and fixed with 4% (v/v) paraformaldehyde and 0.075% (v/v) glutaraldehyde in PBS for 20 min at 37 °C. Parasites were subsequently stained with 5 μM BODIPY TR Ceramide overnight. Coverslips were mounted to glass slides using ProLong Glass Antifade Mountant with NucBlue (Hoechst 33342) and allowed to cure. Z-stacked images were captured with a Zeiss LSM 780 confocal microscope with a 100× oil immersion objective, with z-steps of 388.75 nm. Membranous puncta were enumerated using CellProfiler (https://cellprofiler.org/).

### β-Hematin Assay

The extent to which β-hematin formation is inhibited was evaluated using the Nonidet P-40 (NP-40) assay to determine compound IC_50_ values.^32,67,68^ Stock solutions (10 mM) of the test compounds and a chloroquine diphosphate control were prepared in DMSO and water, respectively. The test and control compounds were added in duplicate to the NP-40 detergent (305.5 μM) and serially diluted into a 7:2:1 (v/v) solution of dH_2_O: NP-40­(305.5 μM): DMSO. Heme (25 mM in DMSO, 178.8 μL) was suspended in 1 M acetate buffer (pH 4.8) and 50 μL added to each well, and incubated for 5 h at 37 °C. Following this, 16 μL of a pyridine solution (5:2:2:1 (v/v) pyridine:water:acetone:HEPES buffer (2 M, pH 7.4)) and 30 μL of acetone was added, and absorbance measured at 405 nm. Sigmoidal does-response curves were generated in Graph Pad Prism 6.0.

### Molecular Modeling of *P. falciparum* PRMTs and Docking of Inhibitors

A homology model of *P. falciparum* PRMT1 was created in the Prime module of Maestro version 13.6.122 by retrieving the *Pf*PRMT1 FASTA sequence from PlasmoDB. A model of *Pf*PRMT1 was created based on *Hs*PRMT1 (PDB accession code 8Z2Z, sequence identity 46%, backbone RMSD 0.151 Å) with complete coverage of the catalytic fold. The model was processed using the Protein Preparation Wizard module of Maestro applying the OPLS-2005 force field to add hydrogens and perform a restrained energy minimization to resolve steric clashes. Receptor grids were generated centered 10 Å in all dimensions around the SiteMap sites. Ligands were prepared using the LigPrep module of Maestro to energy minimize each structure and generate isomeric and tautomeric variants. TC-E 5003 was docked to the homology model using induced-fit docking, and analogs were docked using the extra precision mode of Glide with the TC-E 5003 top-ranked pose as a core constraint. Docking modes were refined, scored, and rank ordered by binding free energy using Prime MM-GBSA.

### Molecular Dynamics Simulation of TC-E 5003 Bound to *Pf*PRMT1

A 20 ns molecular dynamics simulation was performed using Desmond.^69^ The system was constructed by using the OPLS4 force field in the System Builder module. Ligand-Protein dimer complex was solvated with an orthorhombic TIP3P water box with a buffer of 10 Å in every direction. The system was neutralized by the addition of 16 Na+ counterions and supplemented with 0.15 M NaCl to resemble the physiological ionic strength. The model was relaxed before simulation by using the default relaxation protocol, including energy minimization and restrained equilibration. The simulation was performed in the NPT ensemble at 300 K and a pressure of 1.01325 bar. Trajectory frames were recorded every 10 ps, and energy data were recorded every 1 ps. To identify key molecular interactions, interaction analyses were evaluated by using the Simulations Interaction Diagram module.

### Pharmacophore Analysis

The pharmacophore hypothesis for active TC-E 5003 analogs was developed using the PHASE model of Maestro.^38^ Ligand structures prepared using LigPrep and docked with the induced fit binding mode of TC-E 5003 set as a core constraint were imported, and chemical features of the ligands and their cognate *Pf*PRMT1 receptor, prepared above, were defined, with the pharmacophore hypothesis derived from the analogs with pIC_50_ ≥ 5.7 (6 compounds) and inactives as pIC_50_ ≤ 5.0 (13 compounds). Compounds with intermediate activity were excluded (8 compounds). The top ranked model, containing two aromatic ring features, a hydrogen bond donor, a hydrogen bond acceptor and a hydrophobic feature, was selected and validated by screening against a diverse set of decoys obtained from DUD-E with similar physicochemical properties to the actives.

### *Pf*PRMT1 Expression and Purification

*Pf*PRMT1 was codon harmonized and synthesized with a His tag connected by a Tobacco Etch Virus (TEV) protease recognition site and cloned into pET30a­(+) at the NdeI and XhoI restriction sites. Recombinant His-tagged *Pf*PRMT1 was expressed in the BL21 Star (DE3) *E. coli* strain after induction with isopropyl β-d-thiogalactoside (IPTG) for 3 h at 37 °C. Cells were lysed in a B-PER lysis buffer (Thermo Fisher, proprietary composition) with lysozyme and DNase I with 1× cOmplete mini EDTA-free protease inhibitor cocktail (Roche) for 15 min. The soluble fraction was collected by centrifugation at 15 000 × g for 5 min and combined in equal proportion with a Ni-NTA equilibration buffer (20 mM sodium phosphate, 300 mM sodium chloride (PBS) with 10 mM imidazole; pH 7.4) and loaded onto a Ni-NTA column, then washed 5 times with 2 resin bed volumes of PBS with 40 mM imidazole and eluted in PBS with 250 mM imidazole. Purified recombinant PRMT1 was dialyzed 3 times in 100 volumes of 50 mM sodium phosphate buffer, pH 8.0, with 2 mM DTT and 10% glycerol. Protein was snap frozen in liquid nitrogen and stored at −80 °C until use in activity assays. The identity of the protein was confirmed by TIMs-TOF mass spectrometry (CSIR Biosciences).

### *Pf*PRMT1 Inhibition Assay

To evaluate the ability of TC-E 5003 to inhibit recombinant *Pf*PRMT1, a commercial type I PRMT activity and inhibition colorimetric assay kit was used (EpigenTek, all materials proprietary) as per the manufacturer’s instructions. The use of this kit was predicated on the demonstrated type I PRMT activity displayed by *Pf*PRMT1.^18^ To wells coated in a type I PRMT substrate, 100 ng of purified protein, TC-E 5003, and *S*-adenosylmethionine as a methyl donor were added in the PRMT assay buffer. Wells were then incubated for 90 min at 37 °C and then washed with wash buffer. A capture antibody specific to the methylated substrate was added to each well, and the plate was incubated at ambient temperature for 60 min. Unbound antibody was removed, and wells were washed. A detection antibody was added to each well and incubated for 30 min, followed by removal of the unbound antibody and washing of the wells. Developer Solution was added to each well and incubated until the development of blue color. The reaction was stopped with Stop Solution and absorbance was measured at 450 nm.

## Supplementary Material



## Data Availability

All data and materials are available upon reasonable request

## Notes

Figure S1: Structures of diverse PRMT inhibitors evaluated against *P. falciparum*; Figure S2: Activity of TP-064, Onametostat and TC-E 5003 against Dd2 parasites and against HepG2 and CHO mammalian cells; Figure S3: Cumulative parasitemias of treated barcoded mutant parasite pools; Figure S4: β-hematin assay to determine effect of TC-E 5003 on hemozoin formation; Figure S5: Morphological time series of TC-E 5003- TP-064- and Onametostat-treated asexual parasites; Figure S6: Expression profiling of PRMTs in *P. falciparum*; Figure S7: Multiple sequence alignment of PRMT1 protein sequences from *P. falciparum*, *T. gondii*, *H. sapiens* and *R. norvegicus*; Figure S8: Docking mode of TC-E 5003 into *Hs*PRMT1, with overlay of human and parasite binding modes; Figure S9: Expression construct for heterologous expression of *Pf*PRMT1 and SDS-PAGE of expression and purification process of heterologous *Pf*PRMT1; Figure S10: Structures of TC-E 5003 analogues and clustering of analogues by structural similarity, demonstrating clusters of active compounds; Table S1: List of barcoded mutants in the AReBar pool (PDF)

## Abbreviations

ABS: asexual blood stage
AReBar: antimalarial resistome barcoding
CHO: Chinese hamster ovary
PRMT: protein arginine methyltransferase
PTM: post-translational modification
SAH: *S*-adenosyl-l-homocysteine
SAM: *S*-adenosyl-l-methionine
MTOC: microtubule organizing center
Onam.: Onametostat.

## References

[1] World Health Organization . World malaria report 2025; World Health Organization, 2025.

[2] Bozdech Z., Llinás M., Pulliam B. L., Wong E. D., Zhu J., DeRisi J. L. (2003). The transcriptome of the intraerythrocytic developmental
cycle of *Plasmodium falciparum*. PLoS biology.

[3] Martins R. M., Macpherson C. R., Claes A., Scheidig-Benatar C., Sakamoto H., Yam X. Y., Preiser P., Goel S., Wahlgren M., Sismeiro O. (2017). An ApiAP2 member regulates
expression of clonally variant genes of the human malaria parasite *Plasmodium falciparum*. Sci. Rep..

[4] Painter H. J., Campbell T. L., Llinás M. (2011). The Apicomplexan
AP2 family: integral
factors regulating *Plasmodium* development. Molecular and biochemical parasitology.

[5] Connacher J., von Grüning H., Birkholtz L. (2022). Histone Modification Landscapes as
a Roadmap for Malaria Parasite Development. Frontiers in Cell and Developmental Biology.

[6] Connacher J., Josling G. A., Orchard L. M., Reader J., Llinás M., Birkholtz L.-M. (2021). H3K36 methylation
reprograms gene expression to drive
early gametocyte development in *Plasmodium falciparum*. Epigenetics & chromatin.

[7] Cui L., Miao J., Furuya T., Li X., Su X.-z., Cui L. (2007). PfGCN5-mediated histone H3 acetylation
plays a key role in gene expression
in *Plasmodium falciparum*. Eukaryotic
cell.

[8] Jabeena C., Govindaraju G., Rawat M., Gopi S., Sethumadhavan D. V., Jaleel A., Sasankan D., Karmodiya K., Rajavelu A. (2021). Dynamic association of the H3K64 trimethylation mark
with genes encoding exported proteins in *Plasmodium falciparum*. J. Biol. Chem..

[9] Jiang L., Mu J., Zhang Q., Ni T., Srinivasan P., Rayavara K., Yang W., Turner L., Lavstsen T., Theander T. G. (2013). PfSETvs methylation of histone H3K36
represses virulence
genes in *Plasmodium falciparum*. Nature.

[10] Karmodiya K., Pradhan S. J., Joshi B., Jangid R., Reddy P. C., Galande S. (2015). A comprehensive epigenome map of *Plasmodium
falciparum* reveals unique mechanisms of transcriptional regulation
and identifies H3K36me2 as a global mark of gene suppression. Epigenetics & chromatin.

[11] Salcedo-Amaya A. M., van Driel M. A., Alako B. T., Trelle M. B., van den
Elzen A. M., Cohen A. M., Janssen-Megens E. M., van de Vegte-Bolmer M., Selzer R. R., Iniguez A. L. (2009). Dynamic
histone H3 epigenome marking during the intraerythrocytic cycle of *Plasmodium falciparum*. Proc. Natl.
Acad. Sci. U. S. A..

[12] Bártfai R., Hoeijmakers W. A., Salcedo-Amaya A. M., Smits A. H., Janssen-Megens E., Kaan A., Treeck M., Gilberger T.-W., Françoijs K.-J., Stunnenberg H. G. (2010). H2A. Z demarcates intergenic regions
of the *Plasmodium falciparum* epigenome that are dynamically
marked by H3K9ac and H3K4me3. PLoS pathogens.

[13] Lopez-Rubio J.-J., Mancio-Silva L., Scherf A. (2009). Genome-wide analysis
of heterochromatin
associates clonally variant gene regulation with perinuclear repressive
centers in malaria parasites. Cell host &
microbe.

[14] Miao J., Fan Q., Cui L., Li J., Li J., Cui L. (2006). The malaria
parasite *Plasmodium falciparum* histones: organization,
expression, and acetylation. Gene.

[15] Trelle M. B., Salcedo-Amaya A. M., Cohen A. M., Stunnenberg H. G., Jensen O. N. (2009). Global histone analysis
by mass spectrometry reveals
a high content of acetylated lysine residues in the malaria parasite *Plasmodium falciparum*. J. Proteome
Res..

[16] Shrestha S., Lucky A. B., Brashear A. M., Li X., Cui L., Miao J. (2022). Distinct Histone Post-translational Modifications during *Plasmodium falciparum* Gametocyte Development. J. Proteome Res..

[17] von
Grüning H., Coradin M., Mendoza M. R., Reader J., Sidoli S., Garcia B. A., Birkholtz L.-M. (2022). A dynamic
and combinatorial histone code drives malaria parasite asexual and
sexual development. Molecular & Cellular
Proteomics.

[18] Fan Q., Miao J., Cui L., Cui L. (2009). Characterization of
PRMT1 from *Plasmodium falciparum*. Biochem. J..

[19] Fisk J. C., Read L. K. (2011). Protein arginine methylation in parasitic protozoa. Eukaryotic cell.

[20] Fulton M. D., Brown T., Zheng Y. G. (2018). Mechanisms
and inhibitors of histone
arginine methylation. Chem. Rec..

[21] Hwang J. W., Cho Y., Bae G.-U., Kim S.-N., Kim Y. K. (2021). Protein arginine
methyltransferases: promising targets for cancer therapy. Experimental & Molecular Medicine.

[22] Nakayama K., Szewczyk M. M., dela
Sena C., Wu H., Dong A., Zeng H., Li F., de Freitas R. F., Eram M. S., Schapira M. (2018). TP-064, a potent and
selective small
molecule inhibitor of PRMT4 for multiple myeloma. Oncotarget.

[23] Millar H. J., Brehmer D., Verhulst T., Haddish-Berhane N., Greway T., Gaffney D., Boeckx A., Heerde E. V., Nys T., Portale J. (2019). *In vivo* efficacy and
pharmacodynamic modulation of JNJ-64619178, a selective PRMT5 inhibitor,
in human lung and hematologic preclinical models. Cancer Res..

[24] Gerhart S. V., Kellner W. A., Thompson C., Pappalardi M. B., Zhang X.-P., Montes de Oca R., Penebre E., Duncan K., Boriack-Sjodin A., Le B. (2018). Activation of the p53-MDM4
regulatory axis defines the anti-tumour response to PRMT5 inhibition
through its role in regulating cellular splicing. Sci. Rep..

[25] Kim E., Jang J., Park J. G., Kim K.-H., Yoon K., Yoo B. C., Cho J. Y. (2020). Protein arginine methyltransferase
1 (PRMT1) selective inhibitor, TC-E 5003, has anti-inflammatory properties
in TLR4 signaling. International Journal of
Molecular Sciences.

[26] El-Khoueiry A. B., Clarke J., Neff T., Crossman T., Ratia N., Rathi C., Noto P., Tarkar A., Garrido-Laguna I., Calvo E. (2023). Phase 1 study of GSK3368715, a type I PRMT inhibitor,
in patients with advanced solid tumors. British
journal of cancer.

[27] Vieito M., Moreno V., Spreafico A., Brana I., Wang J. S., Preis M., Hernández T., Genta S., Hansen A. R., Doger B. (2023). Phase
1 study of JNJ-64619178, a protein arginine methyltransferase
5 inhibitor, in advanced solid tumors. Clin.
Cancer Res..

[28] Watts J. M., Bradley T. J., Thomassen A., Brunner A. M., Minden M. D., Papadantonakis N., Abedin S., Baines A. J., Barbash O., Gorman S. (2019). A phase I/II study to investigate the safety and clinical
activity of the protein arginine methyltransferase 5 inhibitor GSK3326595
in subjects with myelodysplastic syndrome and acute myeloid leukemia. Blood.

[29] Min H., Lucky A. B., Madsen J. J., Chim-Ong A., Li X., Cui L., Miao J. (2024). Onametostat,
a PfPRMT5 inhibitor, exhibits antimalarial
activity to *Plasmodium falciparum*. Antimicrob. Agents Chemother..

[30] Naude M., van Heerden A., Reader J., van der Watt M., Niemand J., Joubert D., Siciliano G., Alano P., Njoroge M., Chibale K. (2024). Eliminating
malaria transmission requires targeting immature and mature gametocytes
through lipoidal uptake of antimalarials. Nat.
Commun..

[31] Carrasquilla M., Drammeh N. F., Rawat M., Sanderson T., Zenonos Z., Rayner J. C., Lee M. C. (2022). Barcoding genetically
distinct *Plasmodium falciparum* strains for comparative
assessment of fitness and antimalarial drug resistance. Mbio.

[32] Sandlin R. D., Carter M. D., Lee P. J., Auschwitz J. M., Leed S. E., Johnson J. D., Wright D. W. (2011). Use of the NP-40
detergent-mediated assay in discovery of inhibitors of β-hematin
crystallization. Antimicrob. Agents Chemother..

[33] Painter H. J., Chung N. C., Sebastian A., Albert I., Storey J. D., Llinás M. (2018). Genome-wide real-time in vivo transcriptional dynamics
during *Plasmodium falciparum* blood-stage development. Nat. Commun..

[34] Lucky A. B., Wang C., Liu M., Liang X., Min H., Fan Q., Siddiqui F. A., Adapa S. R., Li X., Jiang R. H. Y., Chen X., Cui L., Miao J. (2023). A type II protein arginine
methyltransferase regulates merozoite invasion in *Plasmodium
falciparum*. Communications Biology.

[35] Van
Biljon R., Van Wyk R., Painter H. J., Orchard L., Reader J., Niemand J., Llinás M., Birkholtz L.-M. (2019). Hierarchical transcriptional control regulates *Plasmodium falciparum* sexual differentiation. BMC genomics.

[36] Tewary S. K., Zheng Y. G., Ho M.-C. (2019). Protein arginine
methyltransferases:
insights into the enzyme structure and mechanism at the atomic level. Cell. Mol. Life Sci..

[37] Hendrickson-Rebizant T., Sudhakar S. R., Rowley M. J., Frankel A., Davie J. R., Lakowski T. M. (2024). Structure,
function, and activity of small molecule
and peptide inhibitors of protein arginine methyltransferase 1. J. Med. Chem..

[38] Dixon S. L., Smondyrev A. M., Rao S. N. (2006). PHASE: a novel approach to pharmacophore
modeling and 3D database searching. Chemical
biology & drug design.

[39] Cheng D., Yadav N., King R. W., Swanson M. S., Weinstein E. J., Bedford M. T. (2004). Small molecule regulators
of protein arginine methyltransferases. J. Biol.
Chem..

[40] Saksouk N., Bhatti M. M., Kieffer S., Smith A. T., Musset K., Garin J., Sullivan W. J., Cesbron-Delauw M.-F., Hakimi M.-A. (2005). Histone-modifying complexes regulate gene expression
pertinent to the differentiation of the protozoan parasite *Toxoplasma gondii*. Molecular and cellular
biology.

[41] Coetzee N., von Grüning H., Opperman D., van der Watt M., Reader J., Birkholtz L.-M. (2020). Epigenetic
inhibitors target multiple
stages of *Plasmodium falciparum* parasites. Sci. Rep..

[42] Andrews K., Tran T., Lucke A., Kahnberg P., Le G., Boyle G., Gardiner D., Skinner-Adams T., Fairlie D. (2008). Potent antimalarial activity of histone deacetylase
inhibitor analogues. Antimicrob. Agents Chemother..

[43] Malmquist N. A., Moss T. A., Mecheri S., Scherf A., Fuchter M. J. (2012). Small-molecule
histone methyltransferase inhibitors display rapid antimalarial activity
against all blood stage forms in *Plasmodium falciparum*. Proc. Natl. Acad. Sci. U. S. A..

[44] Matthews K. A., Senagbe K. M., Notzel C., Gonzalez C. A., Tong X., Rijo-Ferreira F., Natarajan B. V., Miguel-Blanco C., Lafuente-Monasterio M. J., Garcia B. A. (2020). Disruption
of the *Plasmodium falciparum* life cycle through transcriptional
reprogramming by inhibitors of Jumonji demethylases. ACS Infectious Diseases.

[45] Nardella F., Halby L., Dobrescu I., Viluma J., Bon C., Claes A., Cadet-Daniel V., Tafit A., Roesch C., Hammam E. (2021). Procainamide–SAHA Fused Inhibitors of hHDAC6
Tackle Multidrug-Resistant Malaria Parasites. J. Med. Chem..

[46] Ngwa C. J., Kiesow M. J., Orchard L. M., Farrukh A., Llinás M., Pradel G. (2019). The G9a histone methyltransferase
inhibitor BIX-01294
modulates gene expression during *Plasmodium falciparum* gametocyte development and transmission. International
journal of molecular sciences.

[47] Reader J., van der Watt M. E., Taylor D., Le Manach C., Mittal N., Ottilie S., Theron A., Moyo P., Erlank E., Nardini L., Venter N., Lauterbach S., Bezuidenhout B., Horatscheck A., van Heerden A., Spillman N. J., Cowell A. N., Connacher J., Opperman D., Orchard L. M., Llinas M., Istvan E. S., Goldberg D. E., Boyle G. A., Calvo D., Mancama D., Coetzer T. L., Winzeler E. A., Duffy J., Koekemoer L. L., Basarab G., Chibale K., Birkholtz L. M. (2021). Multistage and transmission-blocking targeted antimalarials discovered
from the open-source MMV Pandemic Response Box. Nat. Commun..

[48] Vanheer L. N., Kafsack B. r. F. (2021). Activity comparison of epigenetic
modulators against
the hemoprotozoan parasites *Babesia divergens* and *Plasmodium falciparum*. ACS Infectious
Diseases.

[49] Jeninga M. D., Tang J., Selvarajah S. A., Maier A. G., Duffy M. F., Petter M. (2023). *Plasmodium
falciparum* gametocytes
display global chromatin remodelling during sexual differentiation. BMC biology.

[50] Bissinger E.-M., Heinke R., Spannhoff A., Eberlin A., Metzger E., Cura V., Hassenboehler P., Cavarelli J., Schüle R., Bedford M. T. (2011). Acyl
derivatives of
p-aminosulfonamides and dapsone as new inhibitors of the arginine
methyltransferase hPRMT1. Bioorganic & medicinal
chemistry.

[51] El
Bissati K., Suvorova E. S., Xiao H., Lucas O., Upadhya R., Ma Y., Hogue Angeletti R., White M. W., Weiss L. M., Kim K. (2016). *Toxoplasma
gondii* arginine methyltransferase 1 (PRMT1) is necessary
for centrosome dynamics during tachyzoite cell division. MBio.

[52] Liffner B., Cepeda Diaz A. K., Blauwkamp J., Anaguano D., Frolich S., Muralidharan V., Wilson D. W, Dvorin J. D, Absalon S. (2023). Atlas of *Plasmodium falciparum* intraerythrocytic development using
expansion microscopy. Elife.

[53] Langeveld H., Maepa K., Maree M., Thibaud J. L., Salomane N., Bridgwater R., Famodimu M. T., Godoy L. C., Pasaje C. F. A., Boonyalai N. (2025). Targeting Aurora Kinases as Essential
Cell-Cycle Regulators to Deliver Multi-Stage Antimalarials Against *Plasmodium falciparum*. Angew. Chem.,
Int. Ed..

[54] Li J., Shami G. J., Cho E., Liu B., Hanssen E., Dixon M. W. A., Tilley L. (2022). Repurposing the mitotic machinery
to drive cellular elongation and chromatin reorganisation in *Plasmodium falciparum* gametocytes. Nat. Commun..

[55] Godinez-Macias K. P., Chen D., Wallis J. L., Siegel M. G., Adam A., Bopp S., Carolino K., Coulson L. B., Durst G., Thathy V. (2025). Revisiting the *Plasmodium falciparum* druggable genome using predicted structures and data mining. npj Drug Discovery.

[56] Zhang M., Wang C., Otto T. D., Oberstaller J., Liao X., Adapa S. R., Udenze K., Bronner I. F., Casandra D., Mayho M. (2018). Uncovering the essential
genes of the human malaria parasite *Plasmodium falciparum* by saturation mutagenesis. Science.

[57] Oberstaller J., Xu S., Naskar D., Zhang M., Wang C., Gibbons J., Pires C. V., Mayho M., Otto T. D., Rayner J. C. (2025). Supersaturation mutagenesis reveals adaptive rewiring of essential
genes among malaria parasites. Science.

[58] Bushell E., Gomes A. R., Sanderson T., Anar B., Girling G., Herd C., Metcalf T., Modrzynska K., Schwach F., Martin R. E. (2017). Functional profiling
of a *Plasmodium* genome reveals an abundance of essential
genes. Cell.

[59] Cevenini L., Camarda G., Michelini E., Siciliano G., Calabretta M. M., Bona R., Kumar T. S., Cara A., Branchini B. R., Fidock D. A. (2014). Multicolor
bioluminescence
boosts malaria research: quantitative dual-color assay and single-cell
imaging in *Plasmodium falciparum* parasites. Analytical chemistry.

[60] Adjalley S. H., Johnston G. L., Li T., Eastman R. T., Ekland E. H., Eappen A. G., Richman A., Sim B. K. L., Lee M. C., Hoffman S. L. (2011). Quantitative
assessment of *Plasmodium
falciparum* sexual development reveals potent transmission-blocking
activity by methylene blue. Proc. Natl. Acad.
Sci. U. S. A..

[61] Reader J., Botha M., Theron A., Lauterbach S. B., Rossouw C., Engelbrecht D., Wepener M., Smit A., Leroy D., Mancama D. (2015). Nowhere to hide: interrogating
different metabolic parameters of *Plasmodium falciparum* gametocytes in a transmission blocking drug discovery pipeline towards
malaria elimination. Malaria journal.

[62] Reader J., van der Watt M. E., Birkholtz L.-M. (2022). Streamlined and robust stage-specific
profiling of gametocytocidal compounds against *Plasmodium
falciparum*. Frontiers in Cellular and
Infection Microbiology.

[63] Istvan E. S., Mallari J. P., Corey V. C., Dharia N. V., Marshall G. R., Winzeler E. A., Goldberg D. E. (2017). Esterase mutation is a mechanism
of resistance to antimalarial compounds. Nat.
Commun..

[64] Boes A., Spiegel H., Voepel N., Edgue G., Beiss V., Kapelski S., Fendel R., Scheuermayer M., Pradel G., Bolscher J. M., Behet M. C., Dechering K. J., Hermsen C. C., Sauerwein R. W., Schillberg S., Reimann A., Fischer R. (2015). Analysis of a Multi-component Multi-stage
Malaria Vaccine Candidate--Tackling the Cocktail Challenge. PLoS One.

[65] Miglianico M., Bolscher J. M., Vos M. W., Koolen K. J. M., de
Bruijni M., Rajagopal D. S., Chen E., Kiczun M., Gray D., Campo B., Sauerwein R. W., Dechering K. J. (2023). Assessment of the drugability of initial malaria infection
through miniaturized sporozoite assays and high-throughput screening. Commun. Biol..

[66] Vos M. W., Stone W. J., Koolen K. M., van Gemert G.-J., van Schaijk B., Leroy D., Sauerwein R. W., Bousema T., Dechering K. J. (2016). A semi-automated luminescence based
standard membrane feeding assay identifies novel small molecules that
inhibit transmission of malaria parasites by mosquitoes. Sci. Rep..

[67] Fitzroy S.-M., Gildenhuys J., Olivier T., Tshililo N. O., Kuter D., de Villiers K. A. (2017). The effects of quinoline and non-quinoline inhibitors
on the kinetics of lipid-mediated β-hematin crystallization. Langmuir.

[68] Sandlin R. D., Fong K. Y., Stiebler R., Gulka C. P., Nesbitt J. E., Oliveira M. P., Oliveira M. F., Wright D. W. (2016). Detergent-mediated
formation of β-hematin: heme crystallization promoted by detergents
implicates nanostructure formation for use as a biological mimic. Cryst. Growth Des..

[69] Bowers, K. J. ; Chow, E. ; Xu, H. ; Dror, R. O. ; Eastwood, M. P. ; Gregersen, B. A. ; Klepeis, J. L. ; Kolossvary, I. ; Moraes, M. A. ; Sacerdoti, F. D. Scalable algorithms for molecular dynamics simulations on commodity clusters. In Proceedings of the 2006 ACM/IEEE Conference on Supercomputing, 2006; p 43. 10.1109/SC.2006.54.

