# Supplementary material for: Protein Arginine Methyltransferase Inhibitors Target Multiple Stages of Plasmodium falciparum Parasites In Vitro
Source: ACS Infect Dis. 2026 Mar 31;12(4):1455–68. doi: 10.1021/acsinfecdis.6c00182 (PMC13077687; doi:10.1021/acsinfecdis.6c00182)
Supplement: Supplementary file 1 [file id6c00182_si_001.pdf]

SUPPLEMENTARY INFORMATION

Protein arginine methyltransferase inhibitors target multiple stages of *Plasmodium falciparum* parasites *in vitro*

Daniel Opperman[1,2], Tayla Rabie[1,2], Marché Maré[1,2], Mariska Naude[1,2], Mariette van der Watt[2], Jessica L. Thibaud[3], Megan Shannon[3], Nicole Sanders[3], Judith M. Bolscher[4], Rianne van der Laak[4], Rowy Willemsen[4], Alfred Bronkhorst[4], Nonlawat Boonyalai[5], Marcus C. S. Lee[5], Lyn-Marié Birkholtz[1,2,3*]

[1] Department of Biochemistry, Genetics and Microbiology, University of Pretoria, Private bag X20, Hatfield, Pretoria, South Africa

[2] Institute for Sustainable Malaria Control, University of Pretoria, Private bag X20, Hatfield, Pretoria, South Africa

[3] Department of Biochemistry, Stellenbosch University, Stellenbosch, 7600, South Africa

[4] TropIQ Health Sciences, Transistorweg 5, 6534AT Nijmegen, The Netherlands

[5] Division of Biological Chemistry and Drug Discovery, Wellcome Centre for Anti-Infectives Research, University of Dundee, Dundee, UK

* Email: lbirkholtz@sun.ac.za, Tel: +27 21 808 5873

SUPPLEMENTARY INFORMATION

Table of contents:

Supplementary Figures

Figure S1 – S10

Supplementary Table

Supplementary Table S1

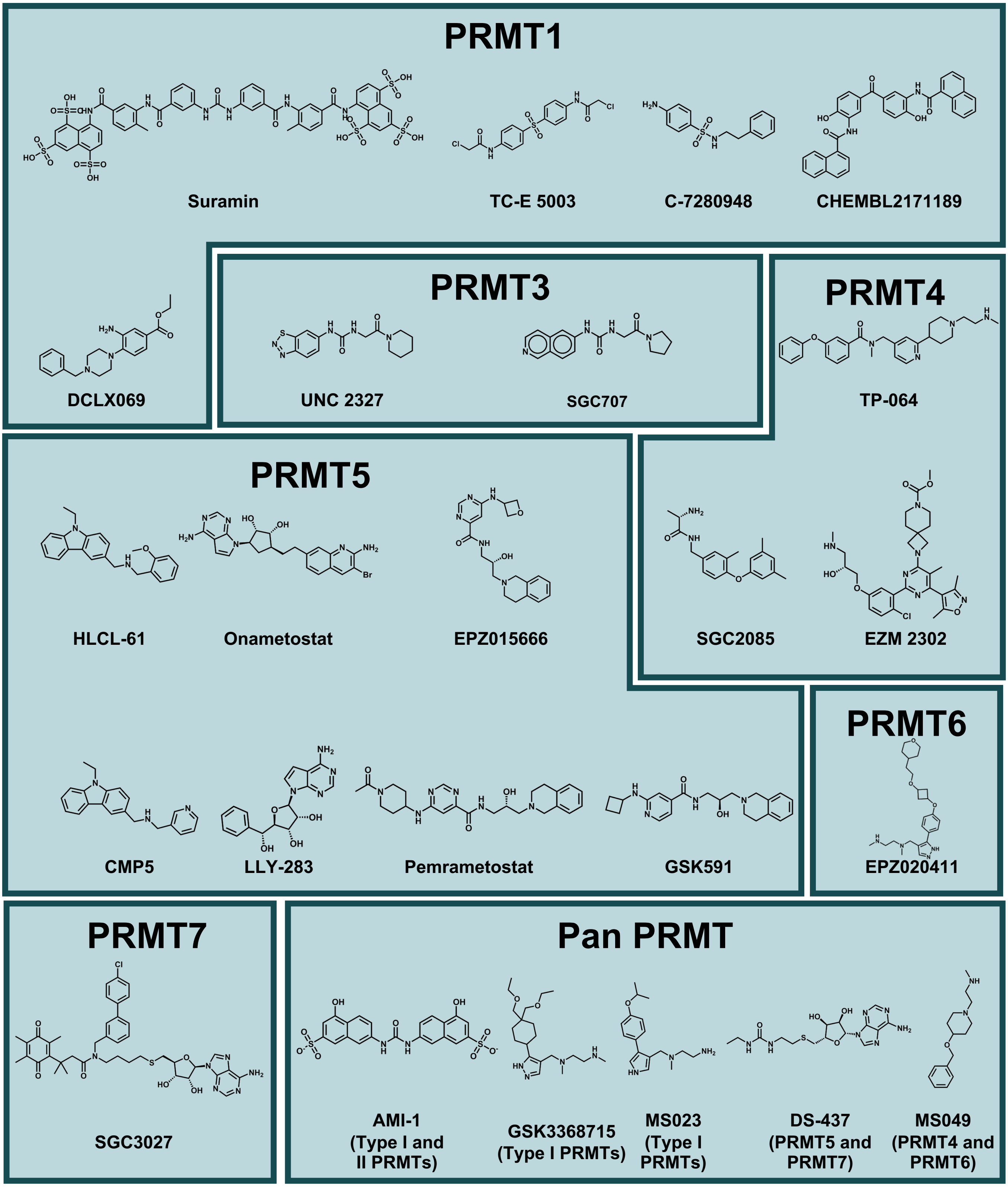

Figure S1: Structures of the selected PRMT inhibitors, grouped by PRMT family member targeted

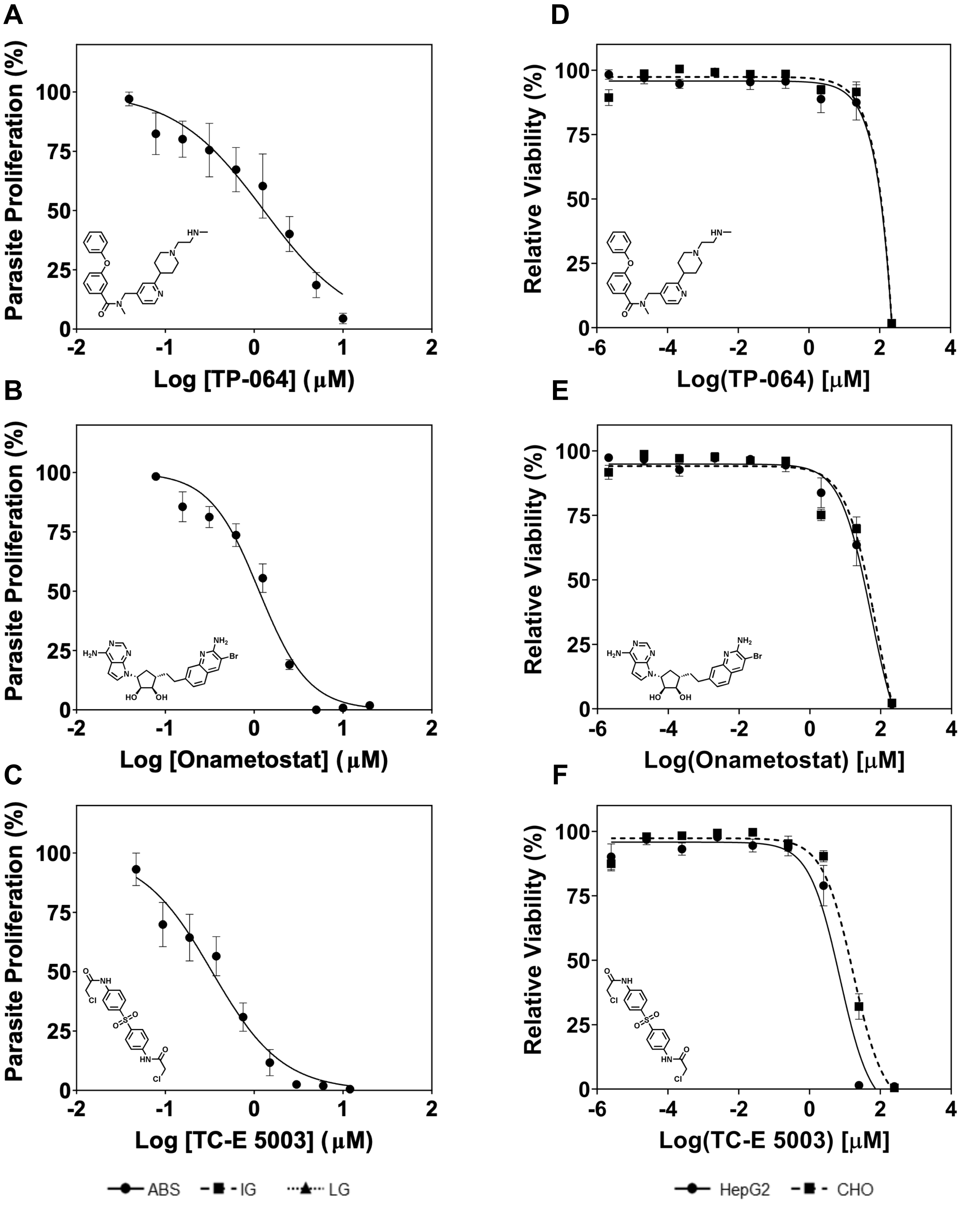

Figure S2: Protein arginine methyltransferase inhibitors show activity against drug-resistant Dd2 *P. falciparum* strain parasites comparable with that against drug-sensitive NF54 strain parasites. Asexual parasites were treated with (A) TP-064, (B) Onametostat or (C) TC-E 5003 for dose-response analyses. TP-064 (D), Onametostat (E) and TC-E 5003 (F) were also tested against HepG2 cells (solid line, circles) and CHO cells (coarse dashed line, squares) to ascertain selectivity for *P. falciparum* cells. Data from three independent biological repeats (n = 3, mean ± S.E.M. indicated).

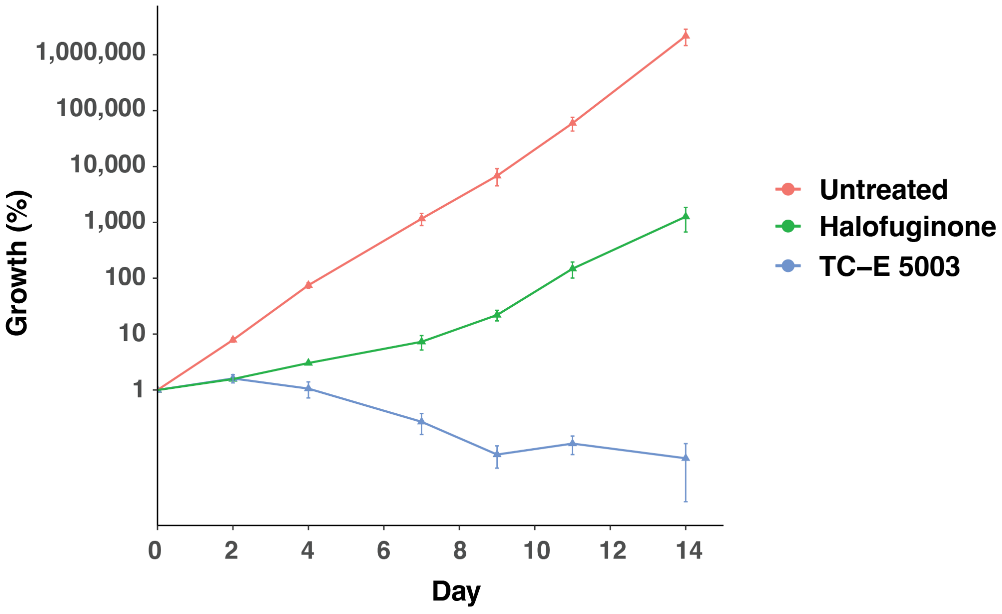

Figure S3: AReBar cross-resistance profiling of TC-E 5003. The cumulative parasitemia of the barcoded parasite pool of mutants, either untreated or exposed to $3xIC_{50}$ of either TC-E 5003 or the proline tRNA synthetase inhibitor halofuginone. Data are from n = 3, mean ± S.D.

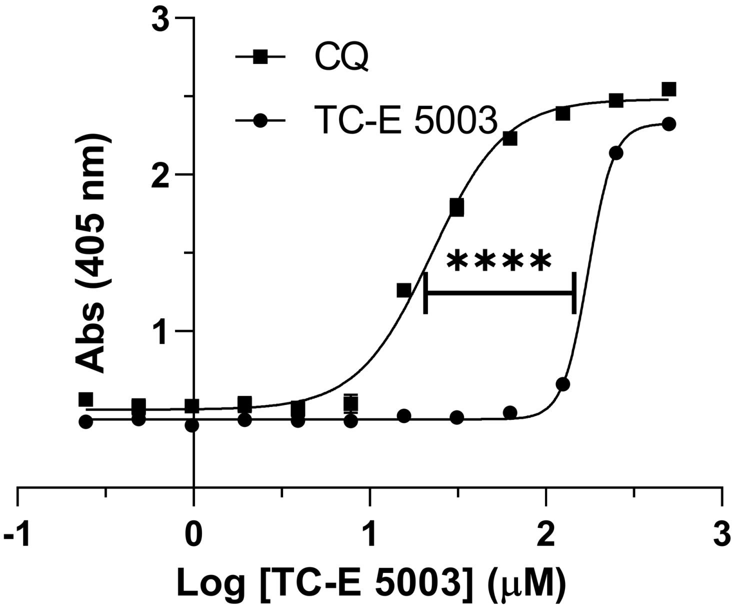

Figure S4: β-hematin assay as a proxy for hemozoin formation. Data are from three biological repeats (n=3), ± S.E.M. Extra sum-of-squares F test was performed. *P*<0.0001.

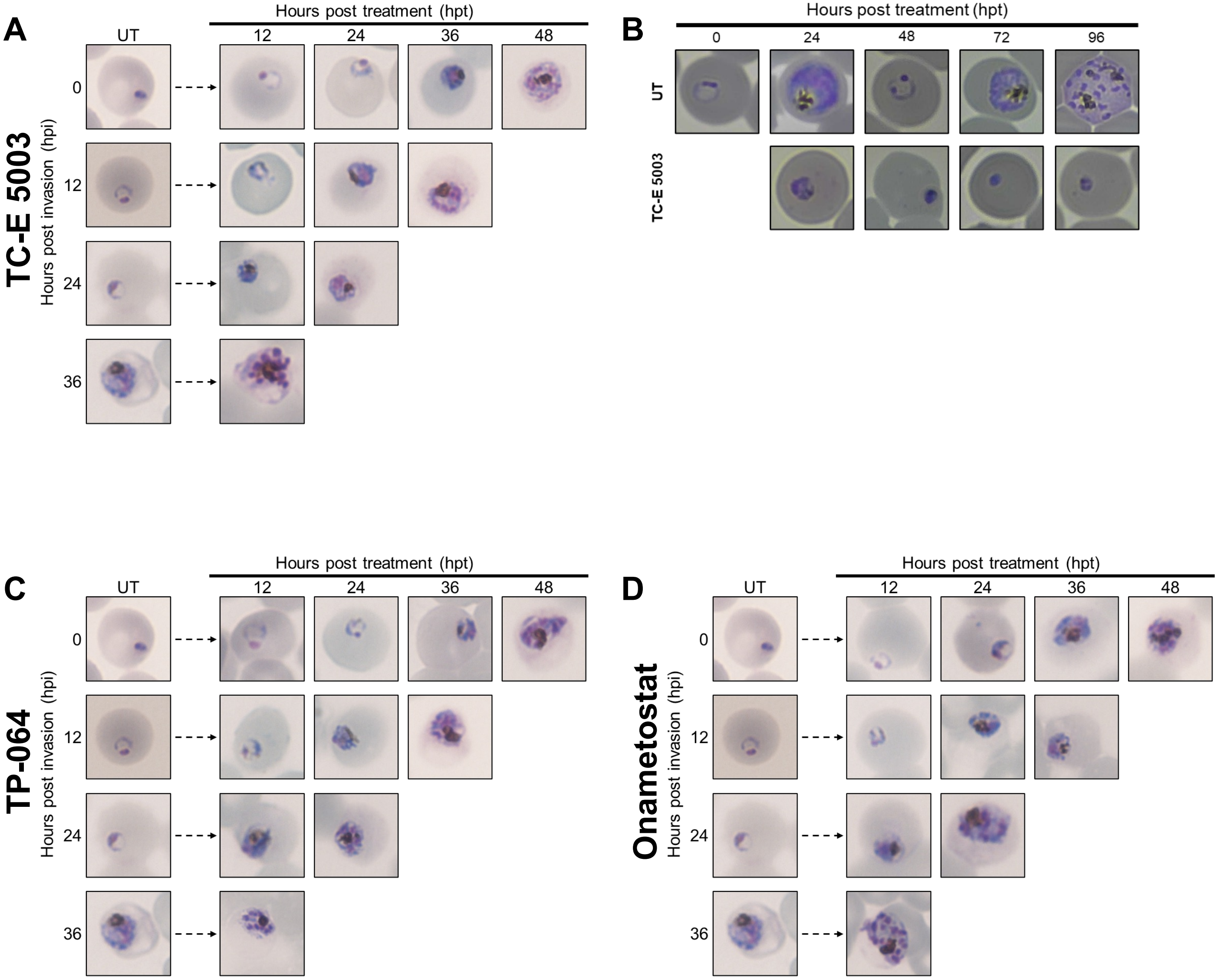

Figure S5: PRMT inhibitors adversely affect schizogony in malaria parasites. Morphologies observed in asexual NF54 strain malaria parasites treated with compounds at 3×IC_{50} at 12, 24, 36 and 48 hours post-treatment with (A) TC-E 5003, (C) TP-064 and (D) Onametostat. (B) Asexual NF54 strain malaria parasites treated at 3×IC_{50} for 96 h.

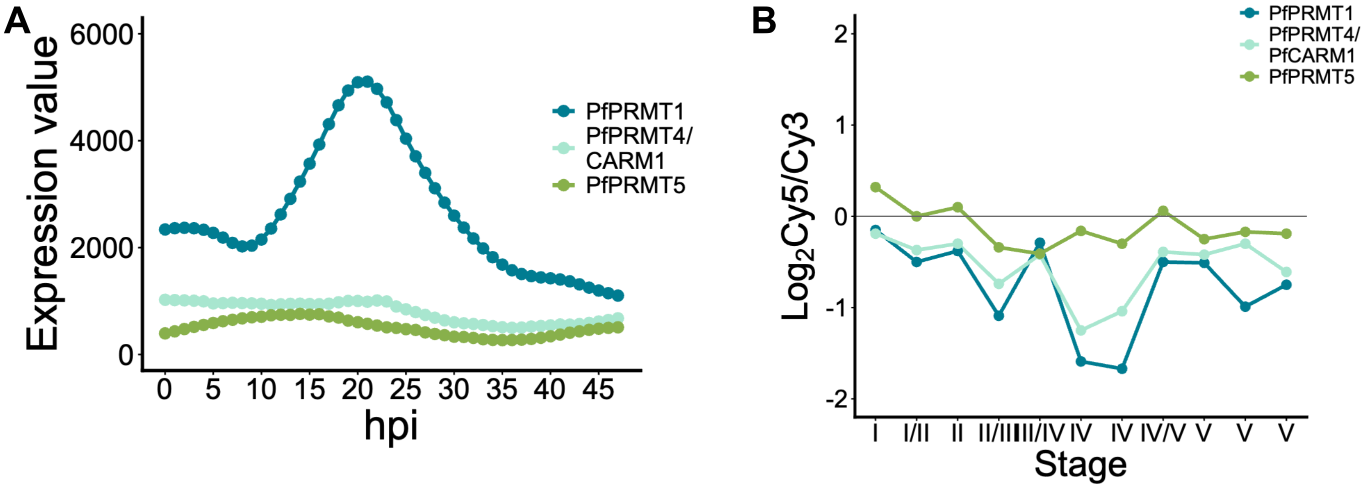

Figure S6: PRMT transcript expression profiles in (A) ABS (Painter *et al. 2012)* and (B) gametocytes (Van Biljon *et al.* 2019) $Log_2Cy5/Cy3$, log_2 of the ratio of Cy5-labelled sample to Cy3-labelled reference pool. Data taken from PlasmoDB.org

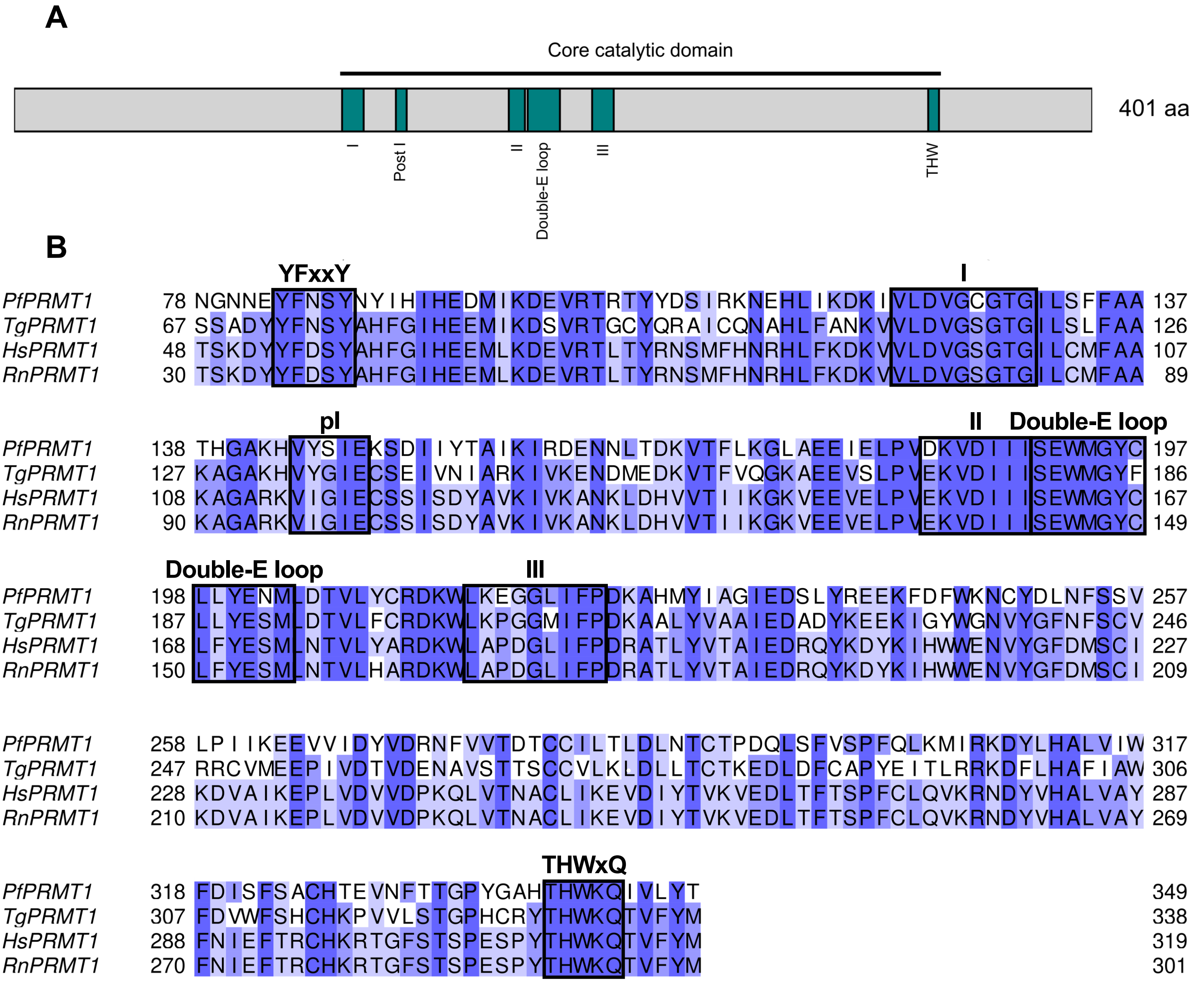

Figure S7: Sequence alignment of catalytic core of PRMT1. (A) Schematic of *Pf*PRMT1 indicating conserved signature catalytic motifs (YFxxY, I, post-I, II, double-E SExMGxxLxxExM loop, III and THWxQ). (B) Alignment of catalytic domains of PRMT1 with YFxxY, I, post-I, II, double-E SExMGxxLxxExM loop, III and THWxQ motifs indicated. Degree of conservation within aligned sequences is indicated by blue shading. *Pf*, *Plasmodium falciparum*; *Tg*, *Toxoplasma gondii*; *Hs*, *Homo sapiens*; *Rn*, *Rattus norvegicus*.

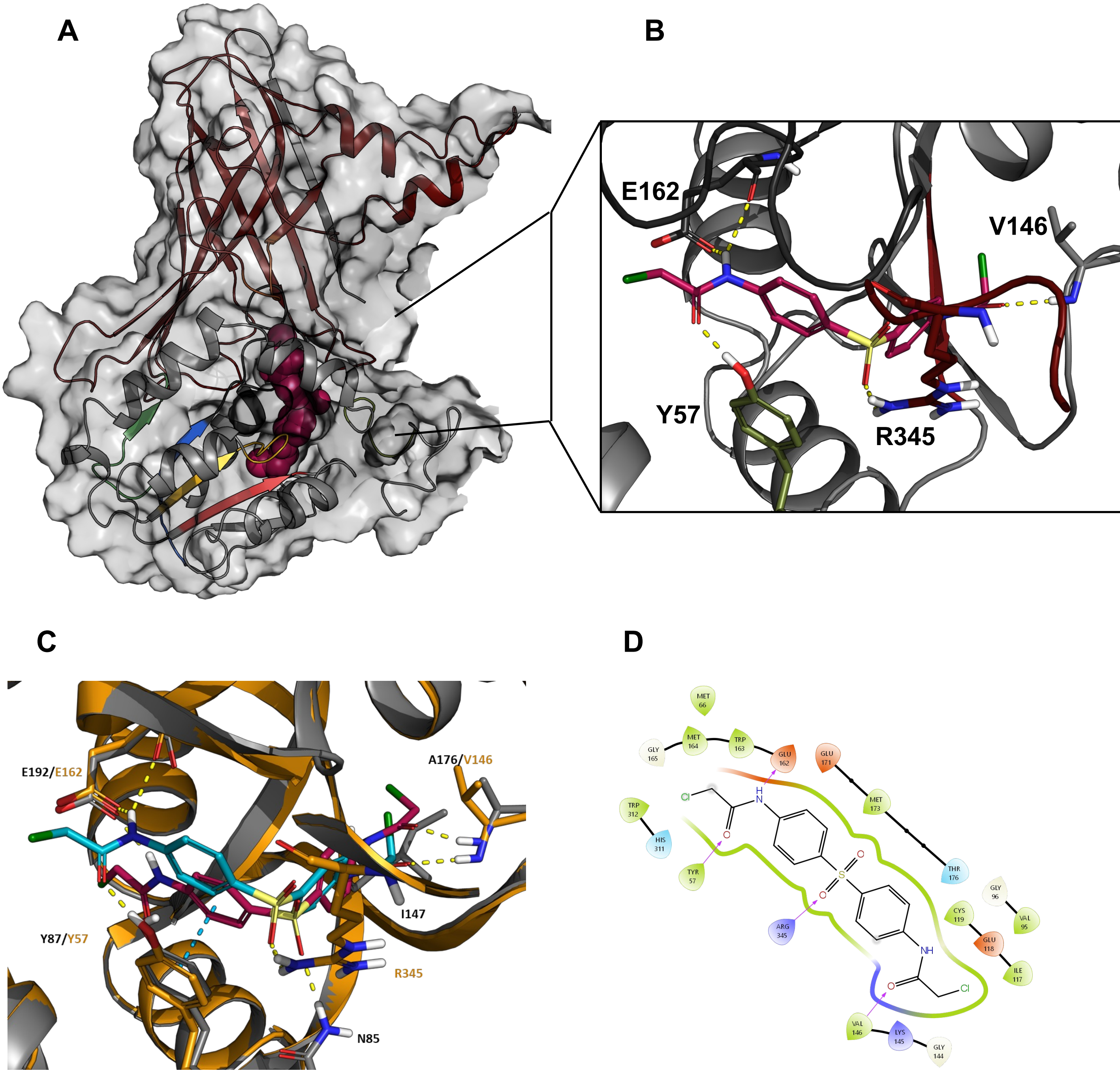

Figure S8: TC-E 5003 docked into crystal structure of human PRMT1, PDB ID 9BHG. TC-E 5003 indicated in magenta. Dimerization domain is shown in deep burgundy with characteristic motifs YFxxY in olive green, I in gold, pI in red, II in blue, double E loop in black, III in green and THW in burnt orange. (A) full view, (B) zoomed view. (C) Overlay of TC-E (magenta) docked into *Pf*PRMT1 (grey, labels in black) and TC-E 5003 (cyan) docked into human PRMT1 (orange, labels in orange). (D) Key interactions between TC-E 5003 and PfPRMT1. Hydrophobic residues shown in green, glycine in yellow, negative in red, positive in blue. Hydrogen bonds indicated in purple. Grey clouds around ligand atoms indicate solvent-exposed regions

A

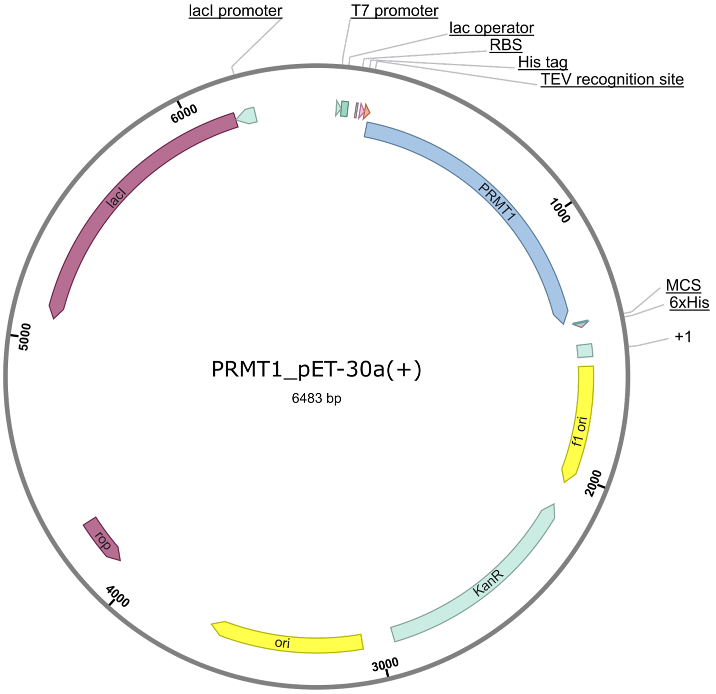

B

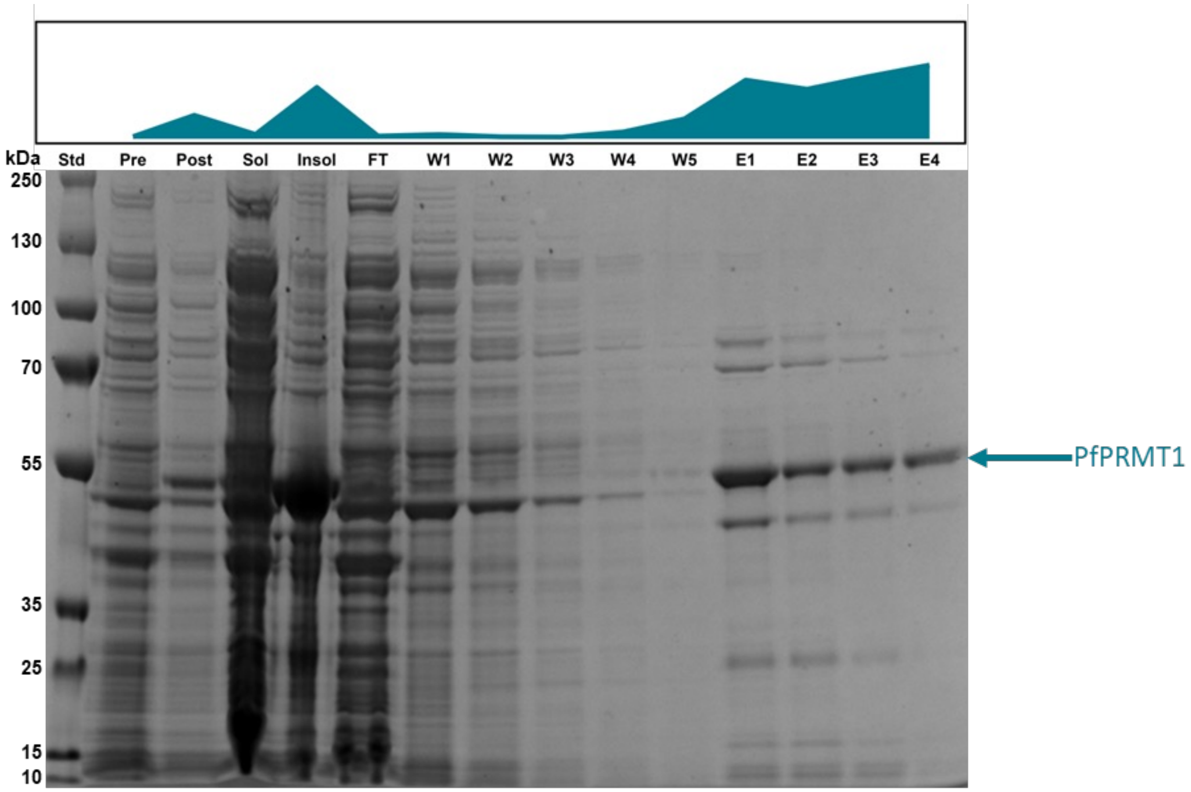

Figure S9: Recombinant expression and purification of PfPRMT1. (A) Expression construct map of PfPRMT1 in pET-30a(+). (B) Std: standards. Pre: pre-induction. Post: post-induction. Sol: soluble. Insol: insoluble. FT: flow through. W: wash. E: elution. Elution fractions 1-3 were pooled. The line graph at the top shows the percentage contribution of PfPRMT1 to the total protein, as determined by densitometric analyses. SDS-PAGE was performed using a NuPage 4-12 % Bis-Tris gel with PageRuler Plus prestained protein ladder. The gel was stained using AcquaStain.

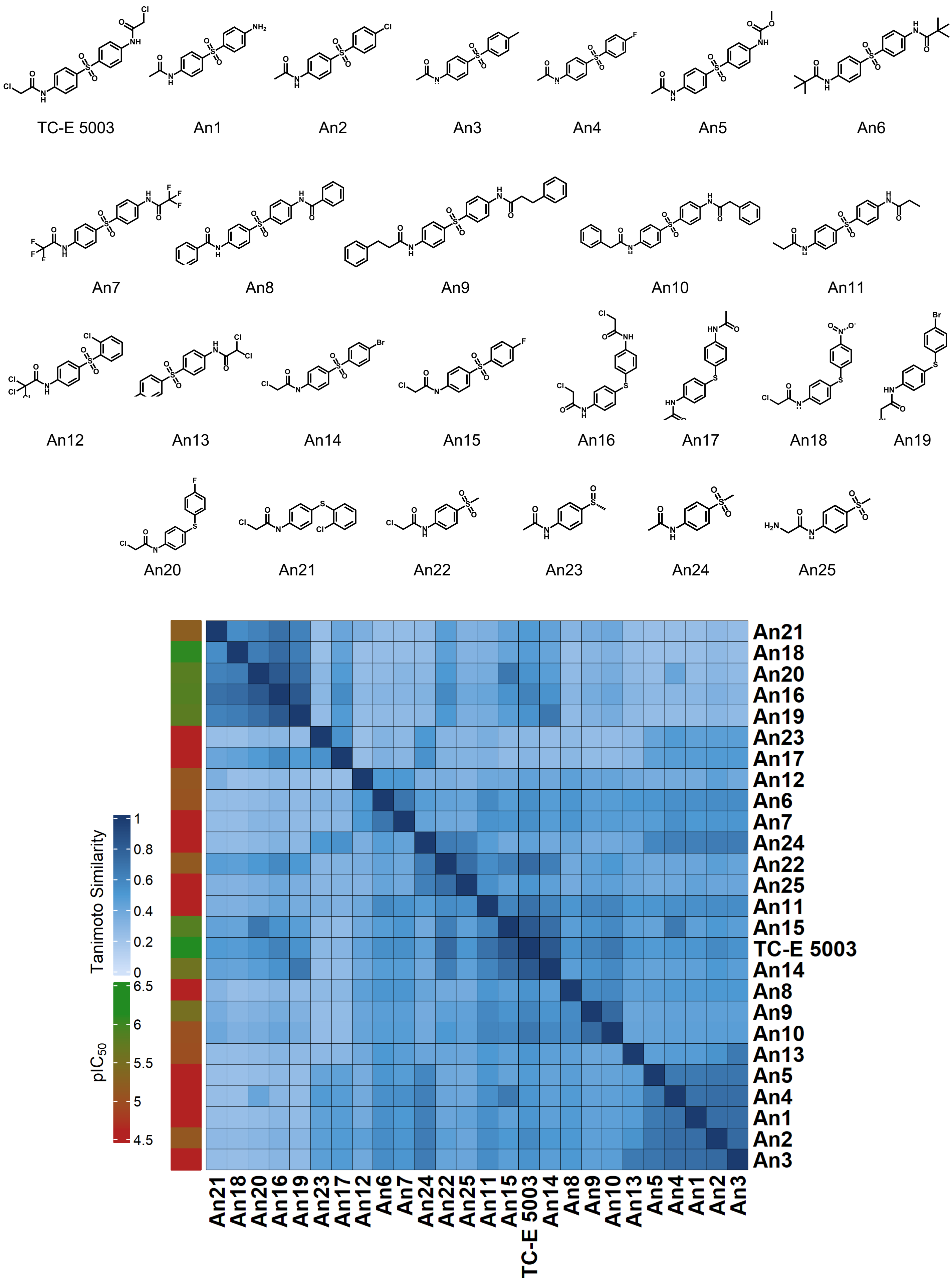

Figure S10: TC-E 5003 analogues evaluated in the hit expansion strategy. Heatmap matrix shows Tanimoto similarity with hierarchical clustering of compounds based on chemical distance in a blue colour scale. Colour bar indicates pIC50 values of individual compounds on a red-green colour scale

Table S1: List of barcoded mutant parasites in the AReBar pool

Line name	Gene Description	Gene ID	Input Day 0	Untreated Day 14	TC-E5003 Day 14
3D7	Wild type		6.11	15.64	7.89
3D7 ABCI3 R2180P	ABC transporter I family member 1	PF3D7 0319700	4.22	3.34	4.44
3D7 ACS10 M300I	Acyl CoA synthase	PF3D7 0525100	6.72	4.56	4.29
3D7 ACS11 D648Y	Acyl CoA synthase	PF3D7 1238800	1.18	0.10	0.45
3D7 ATP2 CNV2	Phospholipid-transporting ATP2	PF3D7 1219600	4.23	2.38	2.84
3D7 DHFR-TS G378E	Dihydrofolate reductase-thymidylate synthase	PF3D7 0417200	1.21	1.12	1.43
3D7 DHFR-TS I403L	Dihydrofolate reductase-thymidylate synthase	PF3D7 0417200	5.02	4.32	4.70
3D7 FTb A515T	Farnesyltransferase subunit beta	PF3D7 1147500	4.31	1.89	2.60
3D7 MDR2 K840N	Multidrug resistance protein 2	PF3D7 1447900	3.45	1.15	3.76
3D7 NCR1 A1108T	Niemann-Pick type C1-related protein	PF3D7 0107500	3.14	2.33	3.49
Dd2	Wild type		3.38	6.16	5.20
Dd2 AcAS A597V	Acetyl-CoA synthetase	PF3D7 0627800	0.44	0.01	0.23
Dd2 AcAS T648M	Acetyl-CoA synthetase	PF3D7 0627800	1.88	2.19	2.11
Dd2 AsnRS R487S	Asn-tRNA synthetase	PF3D7 0509600	1.08	0.74	1.45
Dd2 ATP4 G358S	Non-SERCA-type Ca2+ -transporting P-ATPase	PF3D7 1211900	1.33	0.45	1.14
Dd2 ATP4 L927V	Non-SERCA-type Ca2+ -transporting P-ATPase	PF3D7 1211900	0.93	0.46	1.50
Dd2 ATP4 Q172H	Non-SERCA-type Ca2+ -transporting P-ATPase	PF3D7 1211900	1.79	1.04	1.64
Dd2 CARL I1139K-a	Cyclic amine resistance locus	PF3D7 0321900	0.01	0.00	0.00
Dd2 CARL I1139K-b	Cyclic amine resistance locus	PF3D7 0321900	0.27	0.09	0.17
Dd2 CARL V1103L	Cyclic amine resistance locus	PF3D7 0321900	0.59	0.20	0.42
Dd2 CLK3 H259P	Cyclin-dependent-like kinase	PF3D7 1114700	1.75	1.36	1.72
Dd2 CPSF Y408S E	Cleavage and polyadenylation specific factor	PF3D7 1438500	1.19	0.64	1.11
Dd2 CPSF Y408S S	Cleavage and polyadenylation specific factor	PF3D7 1438500	0.65	0.03	0.58
Dd2 CRT M343L	Chloroquine resistance transporter	PF3D7 0709000	1.74	1.16	1.92
Dd2 CSC1 L800P	CSC1-like protein, putative	PF3D7 1250200	0.70	0.40	0.78
Dd2 cytBC1 G33V	Cytochrome b	PF3D7 MIT02300	1.36	0.03	0.64
Dd2 cytBC1 V284L	Cytochrome b	PF3D7 MIT02300	0.95	0.94	1.38
Dd2 DHFR-TS S216R	Dihydrofolate reductase-thymidylate synthase	PF3D7 0417200	0.91	0.76	1.36
Dd2 DHODH C276Y	Dihydroorotate dehydrogenase	PF3D7 0603300	1.39	1.39	2.21
Dd2 DHODH F227I	Dihydroorotate dehydrogenase	PF3D7 0603300	0.34	0.02	0.33
Dd2 DHODH I263F	Dihydroorotate dehydrogenase	PF3D7 0603300	0.10	0.00	0.08
Dd2 DHODH L531F	Dihydroorotate dehydrogenase	PF3D7 0603300	0.50	0.04	0.33
Dd2 eEF2 L755F	Elongation factor 2	PF3D7 1451100	0.56	0.37	0.73
Dd2 eEF2 Y186N	Elongation factor 2	PF3D7 1451100	0.72	0.07	0.80
Dd2 GGPPS S228T	Geranylgeranyl diphosphate synthase	PF3D7 1128400	0.92	0.50	0.74
Dd2 HSP90 A41S	Heat shock protein 90	PF3D7 0708400	2.10	3.12	2.37
Dd2 IleRS E180D	Ile-tRNA synthetase	PF3D7 1332900	1.63	2.77	3.18
Dd2 IleRS L810F	Ile-tRNA synthetase	PF3D7 1332900	0.87	1.15	1.00
Dd2 IleRS V500A	Ile-tRNA synthetase	PF3D7 1332900	0.79	0.92	0.98
Dd2 kelch13 C580C	Kelch protein K13	PF3D7 1343700	1.68	2.60	2.92
Dd2 kelch13 C580Y	Kelch protein K13	PF3D7 1343700	1.72	1.68	2.31
Dd2 kelch13 R539T	Kelch protein K13	PF3D7 1343700	0.43	0.19	0.56
Dd2 MCP D195N	Mitochondrial carrier protein	PF3D7 0908800	0.38	0.04	0.34
Dd2 MCP P214T	Mitochondrial carrier protein	PF3D7 0908800	4.74	13.00	5.10
Dd2 PI4K S1320L+L1418F	Phosphatidylinositol 4-kinase	PF3D7 0509800	1.50	0.97	1.79
Dd2 PI4K S743F+H1484Y	Phosphatidylinositol 4-kinase	PF3D7 0509800	0.81	0.67	1.08
Dd2 ProRS L482H	Pro-tRNA synthetase	PF3D7 1213800	10.13	2.41	6.02
Dd2 PROTB5 A20V	Proteasome beta 5 26S (A80V immature)	PF3D7 1011400	1.34	0.96	0.57
Dd2 PROTB5 M45I	Proteasome beta 5 26S (M105I immature)	PF3D7 1011400	0.93	0.58	1.31
Dd2 QRP1 D1863Y	Quinoxaline resistance protein	PF3D7 1359900	4.11	11.19	3.52
Dd2 TyrRS S234C	Tyr-tRNA synthetase	PF3D7 1117500	1.43	1.83	2.24
Dd2 UDP-GT F37V	UDP-galactose transporter	PF3D7 1113300	0.35	0.02	0.24
